# Venomics of *Tropidolaemus wagleri*, the sexually dimorphic temple pit viper: Unveiling a deeply conserved atypical toxin arsenal

**DOI:** 10.1038/srep43237

**Published:** 2017-02-27

**Authors:** Choo Hock Tan, Kae Yi Tan, Michelle Khai Khun Yap, Nget Hong Tan

**Affiliations:** 1 Department of Pharmacology, Faculty of Medicine, University of Malaya, 50603 Kuala Lumpur, Malaysia; 2 Department of Molecular Medicine, Faculty of Medicine, University of Malaya, 50603 Kuala Lumpur, Malaysia

## Abstract

*Tropidolaemus wagleri* (temple pit viper) is a medically important snake in Southeast Asia. It displays distinct sexual dimorphism and prey specificity, however its venomics and inter-sex venom variation have not been thoroughly investigated. Applying reverse-phase HPLC, we demonstrated that the venom profiles were not significantly affected by sex and geographical locality (Peninsular Malaya, insular Penang, insular Sumatra) of the snakes. Essentially, venoms of both sexes share comparable intravenous median lethal dose (LD_50_) (0.56–0.63 μg/g) and cause neurotoxic envenomation in mice. LCMS/MS identified six waglerin forms as the predominant lethal principles, comprising 38.2% of total venom proteins. Fourteen other toxin-protein families identified include phospholipase A_2_, serine proteinase, snaclec and metalloproteinase. In mice, HPLC fractions containing these proteins showed insignificant contribution to the overall venom lethality. Besides, the unique elution pattern of approximately 34.5% of non-lethal, low molecular mass proteins (3–5 kDa) on HPLC could be potential biomarker for this primitive crotalid species. Together, the study unveiled the venom proteome of *T. wagleri* that is atypical among many pit vipers as it comprises abundant neurotoxic peptides (waglerins) but little hemotoxic proteinases. The findings also revealed that the venom is relatively well conserved intraspecifically despite the drastic morphological differences between sexes.

Venom is a critical innovation in the evolution of advanced snakes. It represents a trophic adaptive trait crucial for the foraging success of the venomous snakes^1^,^2^. As complex biological products, snake venoms are known to vary across different taxa suited for diverse ecological niches^3^. With the recent advent in proteomic study of snake venoms, the variability in snake venoms is increasingly recognized even within a single species. Intraspecific snake venom variability could be attributed to individual or ontogenic differences^4^,^5^,^6^; however environmental factors (e.g. geographical locale, season) and other endogenous factors (e.g. sex of the snake) are all possible sources of venom variation within the species^7^,^8^,^9^,^10^,^11^,^12^. The primary cause underlying snake venom variability is usually diet and feeding^13^. Diet-driven phenotypic variances are most apparent between adult and juvenile snakes, or between male and female specimens as they have been reported to show differences in prey selection^14^. Presumably, the distinction is due to differences in the body and/or head sizes, where the juvenile and the male (of sexually dimorphic snake) usually feed on smaller prey compared with their counterparts. Unveiling the intraspecific variability of snake venom is hence a crucial component to understand the evolvability and diversity of snake venom toxins. In this context, the Wagler’s or temple pit viper, *Tropidolaemus wagleri* from Southeast Asia is perhaps a model species with intriguing underlying sex-based venom divergence in view of its remarkable sexual dimorphism.

The genus *Tropidolaemus* (Family: Viperidae; Subfamily: Crotalinae), first coined by Wagler in 1830^15^, represents a clade of primitive pit vipers of the Old World. It was subsequently merged into the Asiatic lance-headed pit viper complex (*Trimeresurus sensu lato*) for a long period of time (perhaps since 1842 by Gray)^16^. Morphologically the genus *Tropidolaemus* appears rather different from the rest of *Trimeresurus* species, in which *Tropidolaemus* is characterized by several unique features including the absence of a nasal pore, upper surfaces of the snout, strongly gular keels, head covered with distinctly keeled small scales and the hemipenis type that is closer to Asian basal pit vipers like *Deinagkistrodon acutus, Calloselasma rhodosotma* and *Hypnale hypnale*^17^,^18^. This species has been shown closest to the basal Asiatic pit vipers while diverging earlier from the remaining members of *Trimeresurus* complex, as inferred from molecular and morphological analyses^17^,^18^,^19^,^20^. The genus *Tropidolaemus* was finally resurrected as a distinct genus without close relationship to the genera once contained within the *Trimeresurus* complex^21^, a revolutionary revision that has positively impacted the systematic classification and medical understanding of venom toxicity of this unique group of pit vipers.

*Tropidolaemus* is mainly distributed in Southeast Asia (southern Thailand, southern Vietnam, peninsular Malaya, and many islands including Sumatra, Borneo, Sulawesi and some in the Philippines)^22^. Currently, at least four species of *Tropidolaemus* are recognized^17^, including the most commonly described in the literature and media, the temple pit viper (*Tropidolaemus wagleri*, also known as speckled pit viper and Wagler’s pit viper). *Tropidolaemus wagleri* is an arboreal venomous species that feed mainly on rodents and birds, and occasionally frogs and lizards. Like other *Tropidolaemus* species, both males and females are similarly looking in their juvenile phase but the adult snakes demonstrate considerable sexual dimorphism. The female can grow up to 1 m in total body length and appear bulky. The adult females usually have a black or dark green base coloration with yellow cross-banding, while some variations in coloration do exist. The males basically retain most of the external characteristics from the juvenile phase; they are typically slender and much smaller than the females (Fig. 1). The male body is generally light green in color, ornamented with red and white post-auricular stripes, as well as red and white spots on the dorsum. This species is commonly known as temple pit viper due to its abundance in the Temple of Azure Clouds on the Penang Island, Malaysia. As arboreal predatory ambushers, they are excellent climbers that can remain motionless for a long time – days to weeks - in wait for prey. Being venomous, *T. wagleri* are categorized under Class 2 of medically important snakes by the WHO^23^. The bites are defensive and usually uncommon, but can occur due to careless handling by keepers or agitation brought by workers in plantations including orchards where the snakes hunt for preys. Its bites are known to cause local envenomation (such as pain and swelling) that can be managed conservatively. Thus far, it is unclear whether this snake is able to inflict a fatal bite.

Although the bite rarely leads to severe systemic envenomation, the venom and toxins of this species have been interesting subjects of toxinological research. Unlike most other pit vipers, *T. wagleri* venom does not possess hemorrhagic or anticoagulant activity^24^,^25^. The venom consists of unique glycosylated phospholipase A_2_ and exhibits a range of common enzymatic activities: hyaluronidase, L-amino acid oxidase and phospholipase A_2_, but these are non-lethal in mice^24^,^26^. The lethal components were shown to be the low molecular mass proteins of 3–8 kDa in the venom, with a median lethal dose of approximately 0.2 μg/g in mice^24^. These small proteins were likely the two basic and lethal polypeptides subsequently isolated from the venom, termed waglerin I and waglerin II^27^. Waglerins had been shown to elicit neurotoxic signs in laboratory animals^28^,^29^,^30^.

The uniqueness of *T. wagleri* venom was further demonstrated by the lack of immunological recognition of its toxins by antisera or antivenoms raised against several Asiatic viperid species^31^,^32^. The remarkably different venom properties of this species among *Trimeresurus sensu lato* is congruent with the revised taxonomy where it diverged earlier from the other paraphyletic arboreal pit vipers. Further characterization of its venom is hence important to understand better the biological and medical importance of pit vipers of this unique lineage. Although several in-depth studies on isolated or synthetic waglerins have been reported^27^,^28^,^29^,^30^,^33^, to date, the compositional details of the global venom profile of *T. wagleri* remains unavailable. Confounding this is the lack of insights into venom variability that may accompany the extreme sexual dimorphism of this species. Therefore, this study aimed to investigate the venom proteome of *T. wagleri* by detailing the subtypes and relative abundances of proteins in the venom. Potential divergence in the venom compositions caused by sex factor was also examined. In addition, the proteomic findings were corroborated by toxicity tests for further insights into the toxicovenomic profile of this species.

## Results

### Comparative profiling of *T. wagleri* venoms on reverse-phase HPLC

**Figure 2:**
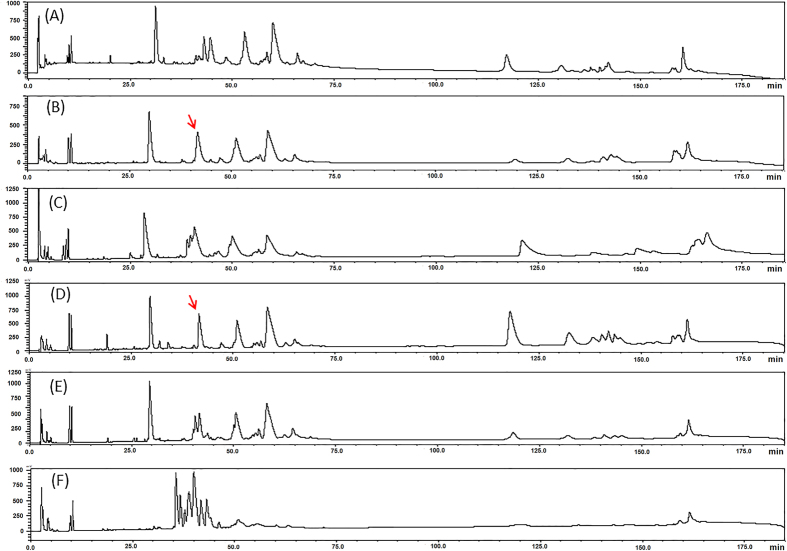
Reverse-phase HPLC of venom samples on Lichrosphere^®^ RP100 C_18_ column. Venoms of *Tropidolaemus wagleri*: (**A**) Perak, female, *n* = 6; (**B**) Perak, male, *n* = 4; (**C**) Penang, female, *n* = 6; (**D**) Penang, male, *n* = 4; (**E**) Sumatra, female, *n* = 6. Venom of *Tropidolaemus subannulatus*: (**G**) Kalimantan, single female for comparison. Arrows indicate the single-peak fraction eluted between 38–42 min for male *T. wagleri* venom.

Figure 2 shows the chromatographic profiles of *T. wagleri* venom according to male and female specimens and their geographical locales. The findings revealed an overall similar elution pattern under the same condition of high performance liquid chromatography for *T. wagleri* venom (Fig. 2A–E), with the exception of the fraction eluted between 38–42 min where the female samples (Fig. 2A,C,E) showed 2–3 neighboring peaks within the eluted fraction, while the male samples (Fig. 2B,D) showed a better resolved single chromatographic peak (indicated by arrows). Another difference was noted in Fig. 2D (Penang, male sample) where the fraction eluted at 120 min appeared higher than that of the other samples. On the other hand, the venom of *Tropidolaemus subannulatus* (included as a hetero-specific sample for profile comparison) exhibited a very different elution profile altogether, as shown in Fig. 2F.

### Venomics of *T. wagleri*

**Figure 3:**
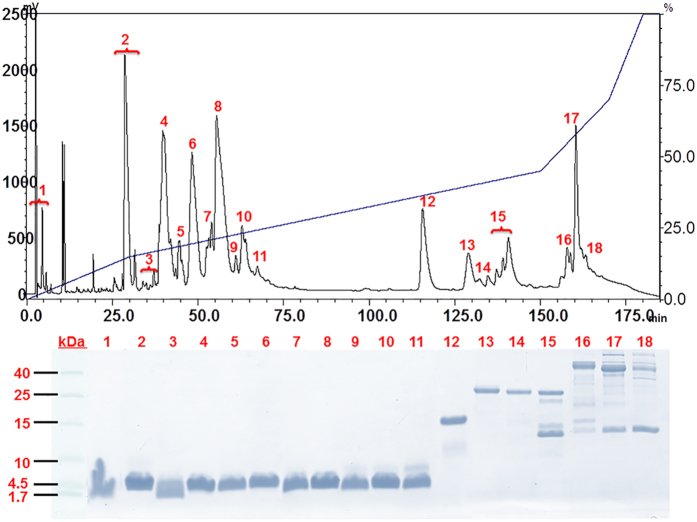
Elution profile of *Tropidolaemus wagleri* crude venom on reverse-phase C_18_ HPLC (upper panel) and further separation by glycine SDS-PAGE (lower panel). Two milligrams of *T. wagleri* venom (from 6 female specimens, Perak, Malaysia) were separated on a Lichrosphere^®^ RP100 C_18_ column following the chromatographic conditions: 5% B for 10 min, 5–15% B for 20 min, followed by 15–45% B for 120 min and 45–70% B for 20 min. The chromatographic fractions were collected manually at 215 nm absorbance and the lyophilized fractions were further electrophoresed on glycine SDS-PAGE (18%, reducing condition). The protein bands were visualized by Coomassie blue staining. Low range protein marker was used as molecular mass standard (from 1.7 to 40 to kDa).

Reverse-phase chromatography resolved the venom into approximately 25 peaks, the majority of which were eluted before 75 min of retention time (comprising 73% of the total peaks area) (Fig. 3a). All eluted proteins were collected manually into 18 fractions designated as Fractions 1–18, respectively. SDS-PAGE of these major fractions revealed the presence of proteins as homogenous bands in the range of 3–7 kDa. Proteins eluted after 110 min showed an increase in heterogeneity and molecular sizes, with masses ranging from 14 kDa to 40 kDa and above (Fig. 3b).

**Figure 4:**
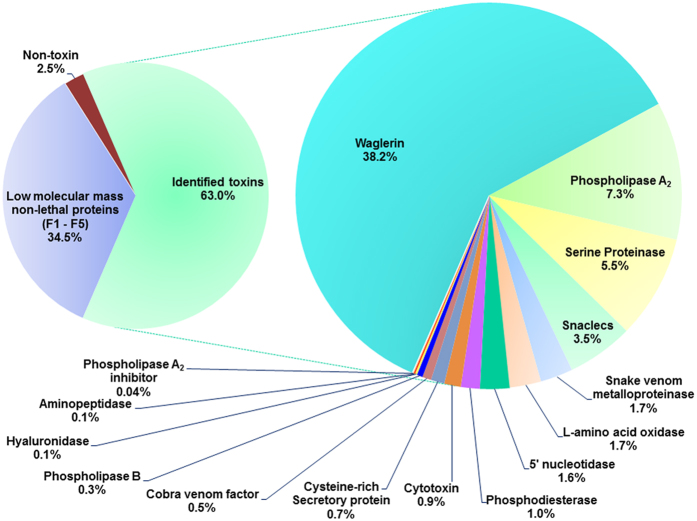
Venom proteome of *Tropidolaemus wagleri* (Perak, Malaysia) venom. Percentage indicates relative abundance of a protein class with respect to total venom proteins.

Liquid chromatography tandem mass spectrometry (LCMS/MS) detected 76 distinct protein forms from Fractions 6–18 of the venom chromatography (Table 1). The proteomic information on mass charges, sequences, validity parameters of the peptide spectra are provided in Supplementary File S1. The in-house transcriptomic data matched by the tryptic peptides were provided in Supplementary File S2. In total, 67 proteins were identified as toxins and assigned into 15 different families (Table 2; Fig. 4). Among these, waglerins were found to be the most abundant (38.2% of total venom proteins), followed by phospholipases A_2_ (7.3%), serine proteinases (5.5%), snaclecs (3.5%), snake venom metalloproteinases (1.7%), L-amino acid oxidases (1.7%), 5′nucleotidases (1.6%), phosphodiesterases (1.0%) and several toxins of lower abundance (<1.0% each) comprising cysteine-rich secretory protein, phospholipase B, hyaluronidase, aminopeptidase, phospholipase A_2_ inhibitor, cytotoxin and cobra venom factor. Together, these toxins constitute 62.3% of the total venom proteins. A small amount of proteins in the venom (2.5%) were identified as non-toxins; these are mainly physiological proteins or cellular enzymes. Meanwhile, venom proteins eluted in the initial course of the chromatography (Fractions 1–5, totaling 34.5% of venom proteins) (Fig. 3a), were assigned as unspecified low molecular mass proteins as they were unidentified by the current LCMS/MS and data mining approach, even with the use of two different protease enzymes (trypsin and chymotrypsin) for optimal sequence matching. Proteins in Fractions 6–11 which share the same low molecular mass as those in Fractions 1–5, nonetheless, were successfully identified as various forms of waglerin.

### Lethality and toxicovenomics of *T. wagleri* venom

The intravenous median lethal doses of the venom in mice were determined to be 0.56 (95% C.I.: 0.37–0.84) μg/g and 0.63 (95% C.I.: 0.59–0.66) μg, respectively, for female and male venom samples (Table 3). Mice injected with a lethal dose of the venom showed prostration and impaired movement with laboured breathing while paralysis set in progressively. Furthermore, the toxicity of the major protein fractions of the venom (Fig. 3a) was evaluated in a mouse model through intravenous injection (Table 4). Of note, Fractions 1–5, unspecified low molecular mass proteins, were not lethal in mice at doses of 1–4 μg/g. Fractions 6 and 8, both being the major waglerin forms, exhibited potent lethality with an LD_50_ value of 0.063 (0.059–0.066) μg/g and 0.20 (0.16–0.25) μg/g, respectively, in mice. On the other hand, Fractions 12–18 which contain proteins of medium to high molecular masses were found to be non-lethal in mice even at high doses (from 2–4 μg/g). For the venom of male specimens, the HPLC fractions eluted between 38–42 min and at 120 min (which showed slight variation from that of the female samples) were also tested in a mouse model. Similarly, these fractions were found to be non-lethal in mice up to 4 μg/g. When tested in frogs, the male and female *T. wagleri* venoms showed comparable neurotoxicity (frogs showed inability to move four limbs) and lethality. The LD_50_ values of female and male venoms are 38.31 (95% C.I.: 29.99–48.94) μg/g and 41.77 (95% C.I.: 32.78–53.23) μg/g, respectively, when injected via lymphatic route into the frogs (Table 3).

## Discussion

Snake venom variability within the same species has been widely appreciated for its implication on fundamental research, management of envenomation and study of venom evolution. Intraspecific venom variability is often associated with geographical origin, age (stage of development), sex, diet etc. of the snake^7^,^10^,^11^,^34^. Recent proteomic approach revealed that there are at least subtle sex-based differences in the venom compositions of some New World pit vipers notably *Bothrops jararaca*^12^,^35^,^36^; however, literature on this comparative topic for Asiatic pit vipers remains scarce. Sex-based venom variation was once reported previously in the Malayan pit viper (*Calloselasma rhodostoma*), although the female-specific protein band (from isoelectric focusing study) was never identified^11^. In most of the studies, the biological significance of sex-based venom variation has not been fully elucidated although it could be sensibly related to dietary differences between male and female snakes. In the present work, the venom variability driven by sexual dimorphism was expected to be much drastic in *T. wagleri* when considering the extreme differences in their body sizes (male-to-female body ratio can be as high as 1:20). Hypothetically, the venom of female *T. wagleri* would be one that is streamlined for the predation of larger animals such as warm-blooded rodents or birds, while the smaller male adults and juveniles feed mainly on amphibians and reptiles. Surprisingly, the current study revealed highly similar chromatographic profiles of both the male and female venoms, in particular the lethal components i.e. waglerins. The findings imply that the venom contents of *T. wagleri* are consistent regardless of the sex and body size of the snake, and that predation is accomplished with the venom neurotoxic activity mediated through waglerins. Functionally, this is further supported by the comparable lethal potency and paralytic effect of male and female venoms in each prey type (mice and frogs). In this study, the mice appeared to be more susceptible than frogs to the lethal effect of both male and female venoms, possibly because waglerin has a higher affinity toward muscle cholinergic receptors of mammalian species.

Essentially, the present work suggested that both male and female *T. wagleri* share the similar composition of lethal principles in their venoms and both induce comparable toxicity. This finding is in agreement with earlier studies which indicated that composition variations in some snake venoms occur at negligible to minor scale between sexes of the same species^37^. On the HPLC profiles, the minor difference noted between 38–42 min of elution time (where splitting of fraction was noted in the female profile) may be considered for the presence of protein isoforms in the pooled female venoms. Proteins eluted in this fraction have low molecular mass similar to waglerins but are likely insignificant medically, since these proteins are non-lethal and did not induce any remarkable sign when injected into the animals at doses more than 5 times of the venom LD_50_. This also applies to the corresponding protein fractions (eluted between 38–42 min and 120 min) of the male specimens where no toxicity was observed at high doses. The majority of these non-lethal, high molecular mass proteins are hydrolytic enzymes that may serve digestive and antibacterial purposes instead of foraging^9^. It should be noted that the interpretation was limited to the individual fractions tested; potential toxicity resulting from synergistic interactions of the different non-lethal fractions remain to be further explored.

The current quantitative proteome of *T. wagleri* venom verified the abundance of waglerins in the venom (close to 40% total venom proteins), which has not been clearly elucidated hitherto in a global profile, although based on toxin-isolation studies the estimated abundance could be, rather inconsistent, ranging from less than 1%^27^ to 8–13%^26^ of total venom proteins. The inconsistency was presumably due to the different methods adopted for protein purification. This present study also demonstrated that waglerins predominate in at least 6 protein forms based on their different degrees of hydrophobic interaction with the C18 column. The two most abundant forms of waglerins isolated from Fraction 6 and Fraction 8 demonstrated high lethal activity with the former being more potent by approximately 4-fold. Our finding is hence in agreement with Weinstein *et al*.^27^ where two lethal, low molecular mass peptides (approximately 3 kDa, termed Lethal I and Lethal II) were isolated from the venom of this species. The lack of lethal effect in the other proteins supported waglerins as the principal lethal toxins of the venom. From the evolutionary and biological aspects, waglerin has been compared to azemiophin, a unique short neurotoxic peptide originated from a primitive viper, *Azemiophs feas*, where both toxins share a homologous C-terminal hexapeptide and block nicotinic receptors, but azemiophin is distinct by not possessing disulfide bridges^38^. Waglerins are also proline-rich short peptides akin to azemiophin and other snake venom metalloproteinase propeptides originated from the colubrid *Psammophis mossambicus*^39^. Of note, the proline-rich property creates a “kinky” peptide orientation which probably resisted tryptic digestion of waglerin initially in this study. This was overcome by the subsequent use of chymotrypsin and the corresponding enzyme-specific database search engine that enabled the identification of waglerins in the proteome.

The abundance and protein type of phospholipases A_2_ revealed in the current proteomic study are consistent with previous toxin isolation studies by Weinstein *et al*.^27^ and Tsai *et al*.^26^. Clinically, the effects of *T. wagleri* PLA_2_ on platelet dysfunction (acidic PLA_2_) or myonecrosis (basic K49-PLA_2_) have not been reported. In laboratory mice, the present study confirmed that these PLA_2_ were non-lethal, supporting the observation reported by Weinstein *et al*.^27^. Instead, the role of the PLA_2_ may be related to ancillary function for the digestion of prey tissues. In addition, the current study also reported the presence of three snaclec subtypes (C-type lectins) in the venom with species-specific sequences matched to *T. wagleri* transcriptome database, implying species uniqueness in the peptide sequences of these snaclecs. Snaclecs are toxins responsible for platelet aggregation or disintegration; however, thrombocytopaenia has not been reported clinically in humans, thus suggesting that the action of *T. wagleri* snaclecs may be species-specific or prey-restricted.

The composition of snake venom metalloproteinase (SVMP) is considerably small in *T. wagleri* venom as shown in its proteome. In contrast, the venom proteomes of most Asiatic pit vipers typically charted 20–40% SVMPs that function as hemorrhagins or anticoagulant toxins^10^,^40^,^41^,^42^. Indeed, the venom of *T. wagleri* lacks hemorrhagic effect which is otherwise commonly seen in envenomation by vipers and pit vipers^24^,^25^. On the other hand, the composition of snake venom serine proteinases (SVSPs) including procoagulant enzymes detected in the current study is comparable to that reported for the basal, terrestrial pit vipers in Asia (*Calloselasma rhodostoma, Hypnale hypnale* and *Deinagkistrodon acutus*)^40^,^42^,^43^,^44^. However, the SVSPs and the venoms of these terrestrial pit vipers were known to exhibit much potent procoagulant activity^24^,^45^. There is considerably overlapping of prey between the arboreal *T. wagleri* and these terrestrial pit vipers; nonetheless, in the latter the potent procoagulant effect of venom is a strategy needed for prey subjugation and killing, while *T. wagleri* relies on the neurotoxic action of waglerin for hunting. Again, the feeble procoagulant activity of *T. wagleri* venom is more parallel to the venom effect of Fea’s viper, *Azemiops feas*, which is also non-hemorrhaging but neurotoxic^38^,^46^.

Earlier, the enzymatic activities of phosphodiesterase, phosphomonoesterase, arginine ester hydrolase and L-amino acid oxidase (LAAO) had been detected in *T. wagleri* venom but the abundance of these enzymes were uncharacterized^24^. The current study successfully revealed the composition of these venom enzymes in addition to several other minor components (phospholipase B, hyaluronidase and aminopeptidase) previously not well reported from this venom. These venom enzymes are likely involved in tissue digestion and venom spread, while LAAO may be responsible for anti-microbial effect of the venom^47^. The current proteomic study was also able to detect the minute phospholipase A_2_ inhibitor, the function of which is unknown in venom but be related to the stability of venom storage in the glands. It is also noteworthy that cobra venom factor (a C3 complement factor) and a cytotoxin homologue conventionally found in elapid venoms^7^,^48^ were also detected proteomically as expressed proteins in *T. wagleri* venom, albeit at a small quantity. Further protein isolation and characterization study is hence warranted to fully validate and elucidate the phenomenon.

Meanwhile, it should be noted that the venom samples were collected from wild-caught specimens and each sexual sample (male, female) was matched to the locality. The main sexual samples collected within Malaysia, in total, were from 8 males and 12 females before stratification into the locales of Penang (6 females, 4 males) and Perak (6 females, 4 males). In addition, the Sumatran sample was a pool from 6 female specimens. We acknowledge that the sample size (*n* = 8–12 per sex group) could be a limitation in this study; however importantly, the conclusion was not generated from bias based on a single *T. wagleri* specimen.

## Conclusions

The current study unveiled the unique composition of *T. wagleri* venom in a global profile, addressing the qualitative and quantitative details of the venom proteins. Of note, venoms from both sexes of the snake (paired peninsular and insular collections) demonstrated comparable lethality and protein profile, suggesting that the venom composition is relatively well conserved in the species despite its drastic sexual dimorphism.

## Materials and Methods

### Venoms

Male and female *T. wagleri* from West Malaysia were wild living specimens collected in the State of Perak (Larut and Ipoh from the Peninsular Malaya) and the Island of Penang. The snakes were identified and the venoms were milked according to sex and locality (Perak: 4 male, 6 female; Penang: 4 male, 6 female) into sterile vials by the author CHT. The venoms were lyophilized and stored at −20 °C until further use. Additional venom samples used in the comparative study were pooled venom of adult female *T. wagleri (n* = 6) from central region of Sumatra Island, and the venom of an adult female *Tropidolaemus subannulatus* from Kalimantan, Borneo Island. The collection and the use of snake venoms for research purposes were conducted in accordance with the guidelines and protocols approved by the Institutional Animal Use and Care Committee (IACUC) of the University of Malaya, Malaysia (protocol approval number: #2013-11-12/PHAR/R/TCH).

### Animals

Albino mice (ICR strain, 20–25 g) were supplied by the Animal Experimental Unit, Faculty of Medicine, University of Malaya. Subadult frogs (Rana *sp*.) were obtained from local supplier. The animals were housed and handled according to the guidelines given by the Council for International Organizations of Medical Sciences (CIOMS) on animal experimentation^49^. All methods were carried out in accordance with the guidelines and regulations approved by the Institutional Animal Care and Use Committee of the University of Malaya (protocol approval number: 2014-09-11/PHAR/R/TCH and 2016-190607/TCH/R/PHARM).

### Chemicals and materials

All chemicals and reagents used in the studies were of analytical grade. Ammonium bicarbonate, dithiothreitol (DTT) and iodoacetamide were purchased from Sigma-Aldrich (USA). Mass spectrometry sequencing grade of trypsin and chymotrypsin proteases, Spectra™ Multicolor Low Range Protein Ladder (1.7 to 40 kDa), and HPLC grade solvents used in the studies were purchased from Thermo Scientific™ Pierce™ (USA). LiChroCART^®^ 250-4 LiChrospher^®^ WP 300 RP-18 (5 μm) HPLC cartridge and Millipore ZipTip^®^ C_18_ Pipette Tips were purchased from Merck (USA).

### C_18_ reverse-phase high-performance liquid chromatography (HPLC) and SDS-polyacrylamide gel electrophoresis (SDS-PAGE)

The amount of venom sample subjected to HPLC was 1 mg each for comparative chromatographic profiling and 2 mg (Perak, female specimens) for mass spectrometry study of the protein fractions. Crude venom samples were first reconstituted in ultrapure water and centrifuged at 10,000 g for 5 min. The supernatants were subjected to LiChrospher^®^ WP 300 C_18_ reverse-phase column (5 μm) using a Shimadzu LC-20AD HPLC system (Japan). The venom components were eluted at 1 ml/min with a linear gradient of 0.1% trifluoroacetic acid (TFA) in water as Solvent A and 0.1% TFA in 100% acetonitrile (ACN) as Solvent B, as follows: 5% B for 10 min, 5–15% B for 20 min, followed by 15–45% B for 120 min and 45–70% B for 20 min. In preparation for mass spectrometric analysis, the chromatographic fractions were collected manually at absorbance 215 nm and the lyophilized fractions were further electrophoresed on glycine SDS-PAGE (18%, reducing condition). The protein bands were visualized using Coomassie blue staining. Low range protein marker was used as molecular mass standard (1.7–40 kDa).

### In-solution tryptic/chymotryptic protein digestion and protein identification by tandem mass spectrometry (nano-ESI-LCMS/MS)

The protein fractions from reverse-phase HPLC were subjected to reduction with DTT, alkylation with iodoacetamide, and in-solution digestion with mass-spectrometry grade trypsin or chymotrypsin proteases as described previously^50^. The protease-digested peptides were desalted with Millipore ZipTip^®^ C_18_ Pipette Tips (Merck, USA) according to the manufacturer’s protocol to enhance the performance of mass spectrometry. The peptide eluates were then subjected to nano-electrospray ionization (ESI) MS/MS experiment, respectively. The experiment was performed on an Agilent 1200 HPLC-Chip/MS Interface, coupled with Agilent 6520 Accurate-Mass Q-TOF LC/MS system. Samples were loaded in a large capacity chip 300 Å, C18, 160 nL enrichment column and 75 μm × 150 mm analytical column (Agilent part No. G4240-62010) with a flow rate of 4 μl/min from a capillary pump and 0.3 μl/min from a Nano pump of Agilent 1200 series. Injection volume was adjusted to 1 μl per sample and the mobile phases were 0.1% formic acid in water (A) and 90% acetonitrile in water with 0.1% formic acid (B). The gradient applied was: 3–50% solution B for 30 min, 50–95% solution B for 2 min, and 95% solution B for 5 min, using Agilent 1200 series nano-flow LC pump. Ion polarity was set to positive ionization mode. Drying gas flow rate was 5 L/min and the drying gas temperature was 325 °C. Fragmentor voltage was 175 V and the capillary voltage was set to 1995 V. Spectra were acquired in MS/MS mode with MS scan range of 110–3000 m/z and MS/MS scan range of 50–3000 m/z. Precursor charge selection was set as doubly, triply or up to triply charged state with the exclusion of precursors 922.0098 m/z (z = 1) and 121.0509 (z = 1) set as reference ions. Data was extracted with MH^+^ mass range between 600–4000 Da and processed with Agilent Spectrum Mill MS Proteomics Workbench software packages. Carbamidomethylation of cysteine was set as a single modification. The peptide finger mapping was modified to specifically search against an in-house database that has merged non-redundant NCBI protein sequences of Serpentes (taxid: 8570) with dataset derived the venom-gland transcriptome of *Tropidolaemus wagleri* of a Malaysian origin. Protein identifications were validated with the following filters: protein score >11, peptides score >6 and scored peak intensity (SPI) >60%. The proteins identified were classified as toxins or non-toxins according to their reported putative functions or known toxicity^9^,^48^. The abundance of individual venom toxin was estimated based on its mean spectral intensity (MSI) relative to the total MSI of all proteins identified through the in-solution mass spectrometry, as reported previously^51^,^52^.

### Determination of lethality of *T. wagleri* venoms and toxins

Protein concentrations in the chromatographic fractions were estimated using Thermo Scientific NanoDrop™ 2000/2000 c Spectrophotometers. The animal experiment protocol was based on the Council for International Organizations of Medical Sciences (CIOMS) guidelines on animal experimentation, and was approved by the Institutional Animal Care and Use Committee of the University of Malaya (Ethics clearance numbers: 2014-09-11/PHAR/R/TCH and 2016-190607/TCH/R/PHARM). The median lethal doses (LD_50_) of the venoms and toxin fractions were determined from a serial dose-response study, where the venoms or toxin components were injected intravenously into the caudal veins of mice, or injected into the dorsal lymph sac of frogs. The number of animals tested for each compound was 16–20 (*n* = 4 per dose, 4–5 doses in total). The survival ratio was recorded after 48 h and LD_50_ was calculated using the Probit analysis method^53^.

## Additional Information

**How to cite this article:** Tan, C. H. *et al*. Venomics of *Tropidolaemus wagleri*, the sexually dimorphic temple pit viper: Unveiling a deeply conserved atypical toxin arsenal. *Sci. Rep.* **7**, 43237; doi: 10.1038/srep43237 (2017).

**Publisher's note:** Springer Nature remains neutral with regard to jurisdictional claims in published maps and institutional affiliations.

## Supplementary Material

Supplementary Information

## Figures and Tables

**Figure 1:**
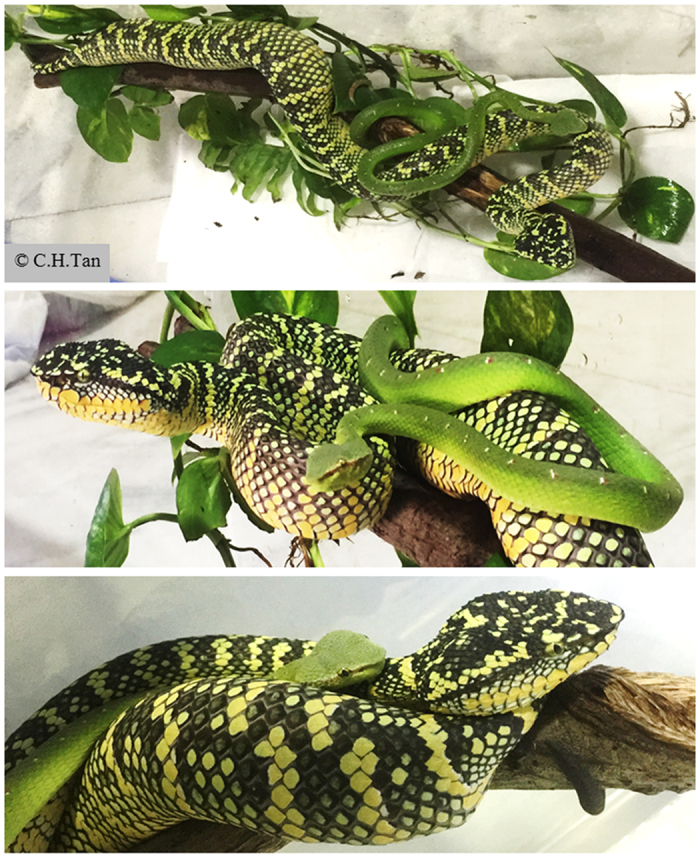
A pair of adult temple pit viper, *Tropidolaemus wagleri* from the Peninsular Malaya, Malaysia. Upper panel: dorsal view; middle panel: lateral view; lower panel: anterior close up view. The snakes are arboreal and both sexes tend to intermingle most of the time as shown in this image. Sexual dimorphism is obvious, from body coloration to size i.e. length and girth of the body, where adult females typically exceed their male counterparts by multiple folds.

**Table 1:** Assignment of *Tropidolaemus wagleri* (Perak, Malaysia) venom proteins by fractions of reverse-phase high performance liquid chromatography.

Fraction	Database Accession	Protein subtypes	Relative abundance (% total venom proteins)
Fraction 6			10.94%
	P24335	Waglerin-3	10.94%
Fraction 7			4.21%
	P24335	Waglerin-3	4.21%
Fraction 8			17.13%
	P58930	Waglerin-4	17.13%
Fraction 9			0.96%
	P58930	Waglerin-4	0.96%
Fraction 10			4.10%
	P58930	Waglerin-4	4.10%
Fraction 11			0.86%
	P58930	Waglerin-4	0.86%
Fraction 12			5.77%
	CL1260.contig1_NKM*	Acidic phospholipase A2 Tgc-E6	4.90%
	Q9W6W9	Cytotoxin 4 N	0.87%
Fraction 13			3.16%
	CL448.contig1_TW*	Serine protease 7	1.99%
	CL448.contig2_TW*	Plasminogen activator-like protein precursor	0.38%
	Unigene24832_TW*	Beta-fibrinogenase mucrofibrase-3	0.78%
	Unigene14693_TW*	Galactose-binding lectin	0.02%
Fraction 14			0.47%
	CL448.contig1_TW*	Serine protease 7	0.11%
	CL448.contig2_TW*	Plasminogen activator-like protein precursor	0.20%
	Unigene14693_TW*	Galactose-binding lectin	0.01%
	CL1654.contig2_TW*	Cysteine-rich secretory protein Og-CRPa	0.00%
	F2Q6F3	Cysteine-rich secretory protein Dr-CRPB	0.00%
	CL1723.contig1_TW*	Basic phospholipase A_2_ homolog acutohaemolysin	0.01%
	Unigene24832_TW*	Beta-fibrinogenase mucrofibrase-3	0.01%
	Unigene24831_TW*	Snake venom serine protease gussurobin	0.07%
	Q71QI7	Snake venom serine protease KN11	0.03%
	Q9YGI6	Snake venom serine protease pallabin-2	0.01%
Fraction 15			**4.92%**
	CL1654.contig2_TW*	Cysteine-rich secretory protein Og-CRPa	0.71%
	CL448.contig1_TW*	Serine protease 7	0.21%
	CL448.contig2_TW*	Plasminogen activator-like protein precursor	0.37%
	CL1723.contig1_TW*	Basic phospholipase A_2_ homolog acutohaemolysin	1.10%
	CL1260.contig2_NKM*	Lys-49 phospholipase A_2_ precursor	1.26%
	Unigene24832_TW*	Beta-fibrinogenase mucrofibrase-3	1.24%
	Unigene14693_TW*	Galactose-binding lectin	0.04%
Fraction 16			2.79%
	Unigene330_TW*	Metalloproteinase isoform 1	0.57%
	CL3502.contig1_TW*	Intestinal-type alkaline phosphatase 1-like TW1	0.16%
	Unigene24659_TW*	Snake venom 5′-nucleotidase	0.25%
	Unigene24655_TW*	Snake venom 5′-nucleotidase	0.26%
	Unigene19683_TW*	GPX3	0.58%
	Unigene14693_TW*	Galactose-binding lectin	0.36%
	CL448.contig1_TW*	Serine protease 7	0.07%
	Unigene22180_TW*	L-amino acid oxidase	0.05%
	CL1504.contig1_TW*	Zinc metalloproteinase-disintegrin HV1	0.04%
	Unigene18_TW*	Agglucetin subunit alpha-2	0.11%
	Unigene23090_TW*	C-type lectin 9	0.30%
	CL1723.contig1_TW*	Basic phospholipase A2 homolog acutohaemolysin	0.02%
	CL2565.contig1_TW*	ADP-ribosyl cyclase 1	0.03%
Fraction 17			7.61%
	Unigene22180_TW*	L-amino acid oxidase	1.48%
	CL2548.contig1_TW*	Phosphodiesterase 1	0.28%
	J3SEZ3	Venom phosphodiesterase 1	0.36%
	Unigene21602_TW*	Phosphodiesterase 1	0.24%
	CL1504.contig1_TW*	Zinc metalloproteinase-disintegrin HV1	0.83%
	Unigene22208_TW*	Gastric intrinsic factor-like	0.47%
	Unigene370_NSM*	Cobra venom factor	0.12%
	CL4560.contig1_NSM*	Cobra venom factor	0.11%
	Q91132	Cobra venom factor	0.16%
	Unigene19683_TW*	GPX3	0.58%
	Unigene22208_TW*	Gastric intrinsic factor-like	0.52%
	Unigene24655_TW*	Snake venom 5′-nucleotidase	0.34%
	Unigene10279_CRM*	Snake venom 5′-nucleotidase	0.30%
	Unigene24659_TW*	Snake venom 5′-nucleotidase	0.29%
	Unigene23090_TW*	C-type lectin 9	0.90%
	Unigene18_TW*	Agglucetin subunit alpha-2	0.27%
	F8S101	Phospholipase B	0.27%
	U3TBU1	Hyaluronidase	0.11%
Fraction 18			2.57%
	CL4560.contig1_NSM*	Cobra venom factor	0.04%
	Q91132	Cobra venom factor	0.04%
	Unigene22180_TW*	L-amino acid oxidase	0.18%
	CL1504.contig1_TW*	Zinc metalloproteinase-disintegrin HV1	0.28%
	Unigene23090_TW*	C-type lectin 9	0.41%
	Unigene19683_TW*	GPX3	0.08%
	Unigene24659_TW*	Snake venom 5′-nucleotidase	0.06%
	Unigene24655_TW*	Snake venom 5′-nucleotidase	0.05%
	Unigene22208_TW*	Gastric intrinsic factor-like	0.05%
	CL3071.contig1_TW*	Aminopeptidase N TW1	0.05%
	Unigene21602_TW*	Phosphodiesterase 1	0.07%
	CL2548.contig1_TW*	Phosphodiesterase 1	0.05%
	Unigene18_TW*	Agglucetin subunit alpha-2	1.10%
	Unigene20823_TW*	Phospholipase B	0.04%
	A5HUI5	Aminopeptidase A	0.02%
	CL2256.contig1_TW*	Phospholipase A_2_ inhibitor beta TW1	0.04%

*Indicate venom proteins identified based on tryptic peptides matched to sequences from in-house transcript-database. Mass spectrometric data and peptide sequences are available in Supplementary File S1.

**Table 2:** Protein family subtypes and relative abundance (%) in the venoms of *Tropidolaemus wagleri*.

Protein family/Protein subtype	Accession No.	Relative abundance (%)
**Toxins**		
**Waglerin**		**38**.**19**
Waglerin-3	P24335	15.15
Waglerin-4	P58930	23.04
**Phospholipase A**_**2**_		**7**.**28**
Acidic phospholipase A_2_ Tgc-E6	CL1260.contig1_NKM*	4.90
Basic phospholipase A_2_ homolog acutohaemolysin	CL1723.contig1_TW*	1.13
Phospholipase A_2_	CL1260.contig2_NKM*	1.26
**Serine Protease**		**5**.**47**
Serine protease 7	CL448.contig1_TW*	2.37
Plasminogen activator-like protein precursor	CL448.contig2_TW*	0.95
Beta-fibrinogenase mucrofibrase-3	Unigene24832_TW*	2.03
Snake venom serine protease gussurobin	Unigene24831_TW*	0.07
Snake venom serine protease KN11	Q71QI7	0.03
Snake venom serine protease pallabin-2	Q9YGI6	0.01
**C-type lectin**		**3**.**50**
Galactose-binding lectin	Unigene14693_TW*	0.42
C-type lectin 9	Unigene23090_TW*	1.60
Agglucetin subunit alpha-2	Unigene18_TW*	1.47
**Snake venom metalloproteinase**		**1**.**72**
Metalloproteinase isoform 1	Unigene330_TW*	0.57
Zinc metalloproteinase-disintegrin HV1	CL1504.contig1_TW*	1.15
**L-amino acid oxidase**		**1**.**71**
L-amino acid oxidase	Unigene22180_TW*	1.71
**5′ nucleotidase**		**1**.**25**
Snake venom 5′-nucleotidase	Unigene24659_TW*	0.60
Snake venom 5′-nucleotidase	Unigene24655_TW*	0.65
Snake venom 5′-nucleotidase	Unigene10279_CRM*	0.30
**Phosphodiesterase**		**1**.**00**
Phosphodiesterase 1	CL2548.contig1_TW*	0.33
Phosphodiesterase 1	Unigene21602_TW*	0.30
Venom phosphodiesterase 1	J3SEZ3	0.36
**Cytotoxin**		**0**.**87**
Cytotoxin 4 N	Q9W6W9	0.87
**Cysteine-rich secretory protein**		**0**.**71**
Cysteine-rich secretory protein Og-CRPa	CL1654.contig2_TW*	0.71
Cysteine-rich secretory protein Dr-CRPB	F2Q6F3	0.00
**Cobra venom factor**		**0**.**47**
Cobra venom factor	Unigene370_NSM*	0.12
Cobra venom factor	CL4560.contig1_NSM*	0.14
Cobra venom factor	Q91132	0.21
**Phospholipase B**		**0**.**31**
Phospholipase B	Unigene20823_TW*	0.31
**Hyaluronidase**		**0**.**11**
Hyaluronidase	U3TBU1	0.11
** Aminopeptidase**		**0**.**08**
Aminopeptidase N TW1	CL3071.contig1_TW*	0.05
Aminopeptidase A	A5HUI5	0.02
**Phospholipase A**_**2**_ **inhibitor**		**0**.**04**
Phospholipase A_2_ inhibitor beta TW1	CL2256.contig1_TW*	0.04
**Non-toxin**		**2**.**46**
GPX3	Unigene19683_TW*	1.23
Gastric intrinsic factor-like	Unigene22208_TW*	1.04
Intestinal-type alkaline phosphatase 1-like TW1	CL3502.contig1_TW*	0.16
ADP-ribosyl cyclase 1	CL2565.contig1_TW*	0.03
**Low molecular mass non-lethal proteins** (**F1–F5**)	—	**34**.**53**

*Indicate venom proteins identified based on tryptic peptides matched to sequences from in-house transcript-database. All peptide sequences are available in Supplementary File S1.

**Table 3:** Median lethal doses (LD_50_) of *Tropidolaemus wagleri* venom from male and female specimens (Malaysia) in mice and frogs (95% C.I. in parenthesis).

*Tropidolaemus wagleri* venom	LD_50_ (μg/g) in mice via intravenous route	LD_50_ (μg/g) in frogs via intra-lymphatic route
Female	0.56 (0.37–0.84)	38.31 (29.99–48.94)
Male	0.63 (0.59–0.66)	41.77 (32.78–53.23)

**Table 4:** Intravenous median lethal doses (*i.v*. LD_50_) of the protein fractions of *Tropidolaemus wagleri* venom (female specimens, Perak, Malaysia) in mice.

Protein Fraction	*i.v*. LD_50_ (95% C.I. in parenthesis) (μg/g)
F1	>4
F2	>1
F3	>1
F4	>4
F5	>2
F6	0.063 (0.059–0.066)
F8	0.20 (0.16–0.25)
F12	>4
F13	>4
F14-15 (pooled)	>4
F16	>2
F17-18 (pooled)	>2

The protein components were resolved by the reverse-phase high performance liquid chromatography.
