# Supplementary material for: Venomics of Tropidolaemus wagleri, the sexually dimorphic temple pit viper: Unveiling a deeply conserved atypical toxin arsenal
Source: Sci Rep. 2017 Feb 27;7:43237. doi: 10.1038/srep43237 (PMC5327433; doi:10.1038/srep43237)
Supplement: Supplementary Information [file srep43237-s1.pdf]

Venomics of *Tropidolaemus wagleri* , the sexually dimorphic temple pit viper: Unveiling a deeply conserved atypical toxin arsenal

Choo Hock Tan, Kae Yi Tan, Michelle Khai Khun Yap, Nget Hong Tan

Supplemetary File S1: QTOF nano-LC-MS/MS of eluted protein fractions from reverse-phase HPLC of *Tropidolaemus wagleri* venom

Fraction	Group	Subgroup	Spectra	Distinct Peptides	Distinct Summed MS/MS Search Score	% AA Coverage	Total Protein Spectral Intensity	Mean Protein Spectral Intensity	Protein MW (Da)	Protein pI	Species	Database Accession	Protein Name	z	Score	Fwd-Rev Score	SPI (%)	Spectrum Intensity	Sequence	RT (min)	Peak Width (sec)	Average Chi Squared	m/z Measured (Da)	Peptide pI	Relative abundance (% in fraction)	Relative abundance (% fraction in chromatogram)	Relative abundance (% overall)
6	1	1.1	1	1	15.67	62.5	9.49E+05	9.49E+05	2836.3	11	*Tropidolae*	P24335	Waglerin-3	3	15.67	15.67	82.2	9.49E+05	(L)GGKPD	4.63	60.97	0.96	597.61	8.61	100.00%	10.94%	10.94%
								9.49E+05																	100.00%		
7	1	1.1	2	1	20.38	66.6	3.89E+06	1.95E+06	2836.3	11	*Tropidolae*	P24335	Waglerin-3	3	14.46	7.49	78.6	3.00E+06	(-)SLGGK	4.53	5.66	0.99	664.32	8.36	100.00%	4.21%	4.21%
														2	20.38	12.73	98.1	8.86E+05	(-)SLGGK	4.52	5.53	0.99	995.97	8.36			
								1.95E+06																	100.00%		
8	1	1.1	4	2	36.65	66.6	2.93E+07	7.33E+06	2862.3	10.43	*Tropidolae*	P58930	Waglerin-4	2	17.74	17.74	93.3	5.45E+06	(L)GGKPD	4.75	11.21	0.99	908.92	8.50	100.00%	17.13%	17.13%
														4	10.3	10.3	75.3	1.51E+07	(-)SLGGK	5.42	22.25	0.98	504.99	8.24			
														3	13.44	8.4	85.7	6.74E+06	(-)SLGGK	5.37	38.85	0.87	672.98	8.24			
														2	18.91	18.91	95.7	2.00E+06	(-)SLGGK	5.40	22.25	0.99	1008.97	8.24			
								7.33E+06																	100.00%		
9	1	1.1	2	1	19.02	66.6	2.82E+06	1.41E+06	2862.3	10.43	*Tropidolae*	P58930	Waglerin-4	3	14.83	9.72	82.7	2.19E+06	(-)SLGGK	5.48	16.72	0.99	672.98	8.24	100.00%	0.96%	0.96%
														2	19.02	19.02	98	6.26E+05	(-)SLGGK	5.47	16.72	0.98	1008.98	8.24			
								1.41E+06																	100.00%		
10	1	1.1	2	2	36.44	66.6	1.58E+07	7.90E+06	2862.3	10.43	*Tropidolae*	P58930	Waglerin-4	2	17.16	17.16	87.7	1.40E+05	(L)GGKPD	5.15	58.94	0.87	908.91	8.50	100.00%	4.10%	4.10%
														2	19.28	19.28	98.3	1.56E+07	(-)SLGGK	5.13	42.34	0.98	1008.98	8.24			
								7.90E+06																	100.00%		
11	1	1.1	3	2	37.47	66.6	4.15E+07	1.38E+07	2862.3	10.43	*Tropidolae*	P58930	Waglerin-4	2	17.89	17.89	95.8	1.00E+07	(L)GGKPD	4.82	22.12	0.99	908.92	8.50	100.00%	0.86%	0.86%
														3	14.6	8.11	87.3	2.23E+07	(-)SLGGK	5.38	27.66	0.95	672.99	8.24			
														2	19.58	19.58	98.3	9.24E+06	(-)SLGGK	5.37	27.66	0.99	1008.98	8.24			
								1.38E+07																	100.00%		
12	1	1.1	4	3	58.59	35	2.16E+06	5.40E+05	15957.8	8.78	*Naja kaou*	60.contig1_	Acidic pho	2	15.41	15.41	78.7	2.05E+05	(K)KYWM	5.50	0.13	0.96	521.77	9.70	27.76%	5.77%	1.60%
														2	17.88	17.88	79.8	2.67E+04	(K)DVYTY	5.68	0.00	0.57	1200.49	4.03			
														3	21.4	21.4	97.7	1.20E+06	(K)DVYTY	6.40	0.00	0.93	795.33	4.03			
														2	21.78	21.78	89.2	7.34E+05	(R)SGIFW	8.25	7.51	0.99	1081.95	8.22			
12	2	2.1	2	2	30.63	23.4	1.93E+05	9.65E+04	9612.5	10.2	*Naja atra*	Q9W6W9	Cytotoxin 4	2	14.72	6.26	88.9	1.57E+05	(K)LVPLF	7.27	0.00	1.00	440.27	8.59	4.96%	5.77%	0.29%
														2	15.91	15.91	80.6	3.58E+04	(K)MFMVS	7.95	5.40	1.00	683.37	8.50			
								6.37E+05																	32.72%		
13	1	1.1	25	8	172.96	43.1	1.15E+08	1.44E+07	28709.3	6.09	*Tropidolae*	48.contig1_	Serine pro	3	17.8	17.8	85.7	2.97E+06	(K)DTCVG	5.28	10.81	0.99	540.91	4.21	62.80%	3.16%	1.99%
														3	19.82	19.82	92	3.65E+06	(K)HIAPLS	6.70	0.13	0.99	654.35	9.75			
														2	15.23	15.23	82.9	1.13E+06	(K)HIAPLS	6.70	0.13	1.00	981.02	9.75			
														2	20.23	10.55	88.2	2.28E+05	(R)FLCSG	6.70	0.13	0.97	646.86	8.75			
														2	19.68	19.68	86.7	6.84E+05	(K)VKLPD	7.58	10.94	1.00	1113.08	5.30			
														2	20.79	20.79	88.4	3.87E+06	(K)LPDVP	7.63	10.94	1.00	999.50	4.41			
														3	25.72	25.72	100	1.06E+07	(R)EFQGL	8.40	16.47	0.96	924.09	4.26			
														2	25.61	25.61	97.2	7.35E+06	(R)EFQGL	8.37	10.93	0.98	1385.64	4.26			
														4	15.1	9.27	89.7	1.94E+05	(R)EFQGL	8.38	10.93	1.00	693.33	4.26			
														3	20.46	20.46	92.9	4.87E+06	(R)AAYPD	8.67	5.66	1.00	746.05	4.14			
														2	27.44	27.44	97.1	3.98E+06	(R)AAYPD	8.67	5.66	1.00	1118.56	4.14			
														4	14.72	14.72	73.4	1.71E+05	(K)DTCVG	8.72	0.13	0.99	1093.75	4.18			
														3	20.48	20.48	78	9.78E+04	(K)DTCVG	8.72	0.13	0.99	1457.99	4.18			
														2	24.84	24.84	97.2	7.41E+06	(R)EFQGL	11.18	203.64	0.99	1385.64	4.26			
														3	26.72	26.72	100	3.08E+07	(R)EFQGL	11.02	190.25	0.99	924.10	4.26			
														3	16.75	16.75	83.5	2.61E+06	(R)AAYPD	10.63	123.20	0.93	746.04	4.14			
														2	16.45	16.45	77.4	8.25E+03	(R)AAYPD	9.87	0.00	0.85	1118.56	4.14			
														2	16.92	16.92	81.8	3.57E+06	(R)EFQGL	12.15	101.11	0.94	1385.64	4.26			
														2	14.41	14.41	87.8	3.56E+06	(R)EFQGL	12.15	100.28	0.94	1385.64	4.26			
														3	24.77	24.77	97	9.42E+06	(R)EFQGL	12.05	76.78	0.99	924.09	4.26			
														2	17.45	17.45	74.5	2.36E+06	(R)EFQGL	12.43	70.74	0.89	1385.64	4.26			
														2	15.7	15.7	78.2	2.32E+06	(R)EFQGL	12.45	69.33	0.89	1385.64	4.26			
														3	22.74	22.74	92.5	1.29E+07	(R)EFQGL	14.53	157.43	1.00	924.09	4.26			
														3	12.06	12.06	73.8	1.10E+04	(R)EFQGL	15.55	0.00	0.89	924.09	4.26			
														3	13.65	13.65	80	8.25E+03	(R)EFQGL	19.60	0.00	0.47	924.09	4.26			
13	1	1.2	4	3	56.51	26.7	8.21E+06	2.74E+06	17432.8	9.52	*Tropidolae*	48.contig2_	Plasminog	3	19.82	19.82	92	3.65E+06	(K)HIAPLS	6.70	0.13	0.99	654.35	9.75	11.96%	3.16%	0.38%
														2	15.23	15.23	82.9	1.13E+06	(K)HIAPLS	6.70	0.13	1.00	981.02	9.75			
														2	20.23	10.55	88.2	2.28E+05	(R)FLCSG	6.70	0.13	0.97	646.86	8.75			
														2	16.46	16.46	72.2	3.20E+06	(R)IMGW(	7.63	5.53	0.98	613.82	8.75			
13	2	2.1	5	3	55.67	23	1.70E+07	5.67E+06	29040.6	7.93	*Tropidolae*	gene24832_	Beta-fibrin	2	17.47	17.47	92.4	2.31E+04	(K)ILDYAV	5.33	0.00	0.96	505.26	5.83	24.76%	3.16%	0.78%
														2	20.09	20.09	92	1.91E+05	(K)VFDYT	7.62	0.13	0.99	593.79	4.21			
														4	17.46	4.73	95.3	1.33E+07	(R)SLVLV	10.37	119.77	0.98	1195.82	4.29			
														5	18.11	5.29	96.8	3.45E+06	(R)SLVLV	9.88	70.66	0.97	956.86	4.29			
														6	17.41	3.36	94.4	6.24E+04	(R)SLVLV	9.25	0.13	0.96	797.55	4.29			
13	3	3.1	2	2	30.02	17.7	2.23E+05	1.12E+05	19250.4	6.51	*Tropidolae*	gene14693_	Galactose	3	15.02	15.02	85.7	2.05E+05	(R)SAESH	5.98	0.13	0.99	626.96	4.72	0.49%	3.16%	0.02%
														2	15	15	89.7	1.77E+04	(K)GQAEV	8.43	0.00	0.99	715.36	4.37			
								2.29E+07																	100.00%		
14	1	1.1	16	12	211.52	63.9	1.91E+07	1.19E+06	28709.3	6.09	*Tropidolae*	48.contig1_	Serine pro	2	11.15	11.15	76.6	2.54E+04	(K)LNEPIS	4.03	0.51	0.82	501.27	6.00	24.00%	0.47%	0.11%
														2	21.27	21.27	80.5	3.10E+05	(K)DTCVG	5.42	0.00	0.99	810.86	4.21			
														2	14.42	4.93	93.7	1.75E+05	(K)NDDEF	5.60	0.00	0.93	802.90	4.36			
														2	13.18	2.86	86.5	2.51E+06	(R)DKDIM	5.73	10.81	1.00	488.28	5.96			
														3	18.31	18.31	91.8	7.06E+06	(K)HIAPLS	6.82	16.21	0.96	654.35	9.75			
														2	15.43	15.43	82.1	2.14E+06	(K)HIAPLS	6.80	5.40	1.00	981.02	9.75			
														3	12.26	12.26	70.8	3.98E+05	(R)FLCSG	6.78	5.40	1.00	431.57	8.75			
														4	18.48	9.3	91.1	3.30E+04	(K)LNEPIS	6.88	5.40	0.96	736.64	8.76			
														3	16.62	8.24	77.4	1.39E+05	(K)WVITA	7.08	27.02	0.99	858.07	6.00			
														2	12.98	12.98	72.6	1.42E+05	(R)AMGW	7.57	0.00	0.98	587.82	8.80			
														3	22.39	22.39	92	1.85E+05	(K)LPDVP	7.83	5.40	0.99	666.67	4.41			
														2	11.21	11.21	70.9	7.72E+03	(K)LPDVP	7.82	0.00	0.65	999.50	4.41			
														3	17.31	17.31	83.9	4.94E+06	(R)AAYPD	8.68	8.52	0.99	746.05	4.14			
														2	24.87	24.87	92.1	4.44E+04	(R)AAYPD	8.40	2.61	0.99	1118.56	4.14			
														3	24.72	24.72	100	7.97E+05	(R)EFQGL	8.47	2.86	1.00	924.10	4.26			
														2	25.59	25.59	96.9	1.92E+05	(R)EFQGL	8.47	2.86	1.00	1385.64	4.26			
14	1	1.2	9	8	120.72	55.5	1.94E+07	2.16E+06	17432.8	9.52	*Tropidolae*	48.contig2_	Plasminog	2	11.15	11.15	76.6	2.54E+04	(K)LNEPIS	4.03	0.51	0.82	501.27	6.00	43.34%	0.47%	0.20%
														2	14.42	4.93	93.7	1.75E+05	(K)NDDEF	5.60	0.00	0.93	802.90	4.36			
														2	13.18	2.86	86.5	2.51E+06	(R)DKDIM	5.73	10.81	1.00	488.28	5.96			
														3	18.31	18.31	91.8	7.06E+06	(K)HIAPLS	6.82	16.21	0.96	654.35	9.75			
														2	15.43	15.43	82.1	2.14E+06	(K)HIAPLS	6.80	5.40	1.00	981.02	9.75			
														3	12.26	12.26	70.8	3.98E+05	(R)FLCSG	6.78	5.40	1.00	431.57	8.75			
														4	18.48	9.3	91.1	3.30E+04	(K)LNEPIS	6.88	5.40	0.96	736.64	8.76			
														3	16.62	8.24	77.4	1.39E+05	(K)WVITA	7.08	27.02	0.99	858.07	6.00			
														2	16.3	16.3	70.7	6.95E+06	(R)IMGW(	7.88	11.39	0.85	613.82	8.75			
14	2	2.1	4	4	69.65	31.6	5.49E+05	1.37E+05	19250.4	6.51	*Tropidolae*	gene14693_	Galactose	3	18.7	18.7	87	2.18E+04	(R)TYKPS	4.58	0.00	0.94	535.26	9.99	2.76%	0.47%	0.01%
														3	15.88	15.88	92.5	2.63E+05	(R)SAESH	6.22	0.00	0.98	626.96	4.72			
														2	15.84	15.84	80.5	1.77E+05	(K)DFSWI	7.50	5.40	1.00	621.27	4.03			
														2	19.23	19.23	90.1	8.68E+04	(K)GQAEV	8.53	3.63	0.99	715.37	4.37			
14	3	3.1	4	4	65.16	26.6	1.26E+05	3.15E+04	27911.9	5.12	*Tropidolae*	654.contig2	Cysteine-r	2	16.56	9.8	84.8	5.30E+04	(R)SVNPT	5.23	0.00	0.97	581.30	8.47	0.63%	0.47%	0.00%
														3	12.96	12.96	86.9	2.50E+04	(K)DIYINC	6.25	5.40	0.93	714.66	4.21			
														2	19.22	19.22	85.1	2.14E+04	(K)MEWY	6.40	0.00	-0.56	769.34	4.25			
														3	16.42	9.18	82.4	2.72E+04	(K)YAVGA	7.92	0.00	0.92	818.08	6.74			

14	3	3.2	3	3	54.76	14.9	1.05E+05	3.50E+04	25956.9	9.33	*Daboia rus*	F2Q6F3	Cysteine-r	2	18.98	11.36	94.7	3.08E+04	(-)SVDFD	4.78	0.00	1.00	569.75	4.03	0.70%	0.47%	0.00%
														2	16.56	9.8	84.8	5.30E+04	(R)SVNPT	5.23	0.00	0.97	581.30	8.47			
														2	19.22	19.22	85.1	2.14E+04	(K)MEWY	6.40	0.00	-0.56	769.34	4.25			
14	4	4.1	5	3	57.51	29.2	6.35E+05	1.27E+05	15775.9	9.55	*Tropidolae*	23.contig1	Basic phos	3	13.79	13.79	74.4	1.39E+05	(K)AIACGI	3.83	3.44	0.98	772.96	4.18	2.55%	0.47%	0.01%
														3	24.69	24.69	93.3	1.93E+05	(K)AIACGI	4.37	3.19	0.93	767.63	4.18			
														2	15.12	15.12	82.7	2.01E+04	(K)AIACGI	4.37	3.19	0.95	1150.94	4.18			
														3	15.5	15.5	87.3	3.56E+04	(K)MDHYS	6.13	0.00	0.99	486.56	6.49			
														2	17.32	7.71	88.6	2.48E+05	(K)TLAICL	6.32	0.00	1.00	438.26	8.41			
14	5	5.1	3	3	55.45	23	2.10E+05	7.00E+04	29040.6	7.93	*Tropidolae*	jene24832_	Beta-fibrin	2	18.84	7.73	95.2	7.64E+04	(K)ILDYAV	5.50	0.00	0.94	505.26	5.83	1.41%	0.47%	0.01%
														2	20.87	20.87	90.2	1.10E+05	(K)VFDYT	7.82	0.00	1.00	593.79	4.21			
														4	15.74	3.39	85.1	2.37E+04	(R)SLVLV	9.28	0.00	0.57	1195.84	4.29			
14	5	5.2	4	3	54.03	25.2	3.03E+06	7.58E+05	28893.6	9.52	*Tropidolae*	jene24831_	Snake ven	2	18.84	7.73	95.2	7.64E+04	(K)ILDYAV	5.50	0.00	0.94	505.26	5.83	15.23%	0.47%	0.07%
														3	18.03	18.03	84.8	7.47E+05	(K)FQAIVS	6.85	10.81	1.00	559.60	9.75			
														2	19.45	15.93	91.1	2.18E+06	(K)FQAIVS	6.83	5.40	1.00	838.90	9.75			
														4	15.74	3.39	85.1	2.37E+04	(R)SLVLV	9.28	0.00	0.57	1195.84	4.29			
14	6	6.1	2	2	34.87	7	6.84E+05	3.42E+05	28740.7	9.3	*Trimeresu*	Q71QI7	Snake ven	2	18.5	13.6	94.3	5.68E+05	(K)TLCAG	5.97	0.00	0.99	559.31	8.41	6.88%	0.47%	0.03%
														2	16.37	16.37	78.6	1.16E+05	(K)FFCLS	6.20	10.55	0.99	444.72	8.75			
14	7	7.1	2	2	33.15	6.1	2.49E+05	1.25E+05	29368.1	5.65	*Gloydius h*	Q9YGI6	Snake ven	2	16.37	16.37	78.6	1.16E+05	(K)FFCLS	6.20	10.55	0.99	444.72	8.75	2.50%	0.47%	0.01%
														2	16.78	10.47	90.7	1.33E+05	(R)FLVAL	7.30	0.00	0.99	535.31	8.75			
								4.97E+06																	100.00%		
15	1	1.1	15	9	216.87	60.8	2.64E+07	1.76E+06	27911.9	5.12	*Tropidolae*	54.contig2	Cysteine-r	2	23.14	23.14	90.4	1.96E+06	(R)MQSY	5.32	0.13	0.95	890.85	8.34	14.35%	4.92%	0.71%
														3	17.19	9.55	81.2	6.45E+05	(K)DIYINC	5.80	5.66	0.97	766.69	5.96			
														2	26.6	26.6	95.1	2.24E+05	(K)DIYINC	5.80	5.66	0.95	1149.53	5.96			
														2	20.73	20.73	90.4	3.10E+06	(K)DIYINC	5.98	5.66	0.98	1071.48	4.21			
														3	15.2	15.2	76.5	1.16E+06	(K)DIYINC	5.97	0.13	1.00	714.66	4.21			
														2	22.6	22.6	88.5	3.79E+06	(K)MEWY	6.07	0.13	0.99	769.34	4.25			
														3	17.14	17.14	82.3	1.02E+04	(K)MEWY	6.15	0.13	0.95	513.23	4.25			
														2	22.03	22.03	95.8	1.33E+06	(R)VVEEI	6.45	5.66	0.98	1107.50	4.25			
														3	16.51	16.51	89.8	1.25E+06	(R)VVEEI	6.45	5.66	0.98	738.67	4.25			
														3	26.7	26.7	100	1.85E+06	(K)SAFPY	6.58	5.66	0.93	1123.78	4.03			
														2	29.95	29.95	100	1.24E+06	(K)SAFPY	6.60	5.66	0.92	1685.17	4.03			
														2	24.44	24.44	97.1	2.86E+06	(R)NPEIQ	7.08	0.13	0.99	953.00	4.65			
														2	20.42	20.42	97.3	4.98E+04	(K)WTEII	7.35	0.13	0.94	848.41	5.40			
														3	21.44	14.16	95.1	5.92E+06	(K)YAVGA	7.72	0.13	0.97	818.08	6.74			
														2	26.96	26.96	96	9.86E+05	(K)YAVGA	7.72	0.13	1.00	1226.62	6.74			
15	2	2.1	14	8	164.25	51.7	7.18E+06	5.13E+05	28709.3	6.09	*Tropidolae*	48.contig1_	Serine pro	2	21.03	21.03	80.4	4.04E+05	(K)DTCVC	5.23	0.13	0.98	810.86	4.21	4.18%	4.92%	0.21%
														3	12.95	12.95	85.4	8.25E+04	(K)DTCVC	5.23	0.13	0.98	540.91	4.21			
														2	19.1	8.76	86.3	8.98E+05	(R)FLCSG	6.63	5.66	1.00	646.86	8.75			
														3	17.99	17.99	88.2	2.05E+06	(K)HIAPLS	6.68	5.66	1.00	654.35	9.75			
														2	15.09	15.09	82	6.84E+05	(K)HIAPLS	6.68	5.66	1.00	981.02	9.75			
														3	17.2	8.98	76.1	2.70E+05	(K)WVITA	6.82	5.66	0.90	858.08	6.00			
														4	13.31	13.31	85.3	2.47E+05	(K)VKLPD	7.60	5.66	0.98	557.05	5.30			
														3	13.76	13.76	72.4	1.24E+05	(K)VKLPD	7.60	5.66	0.97	742.39	5.30			
														3	21.69	21.69	93.2	4.93E+05	(K)LPDVP	7.67	5.67	1.00	666.67	4.41			
														2	22.37	22.37	90.7	5.37E+04	(K)LPDVP	7.65	5.67	0.97	999.50	4.41			
														2	26.35	26.35	97	4.07E+05	(R)EFQGI	8.37	5.15	0.99	1385.64	4.26			
														3	22.08	22.08	77.7	2.68E+05	(R)EFQGI	8.37	5.15	0.98	924.10	4.26			
														2	26.45	26.45	97.2	5.89E+05	(R)AAYPD	8.67	5.40	1.00	1118.56	4.14			
														3	19.73	19.73	93.4	6.06E+05	(R)AAYPD	8.67	5.40	0.99	746.04	4.14			
15	2	2.2	5	4	70.75	41.1	4.65E+06	9.30E+05	17432.8	9.52	*Tropidolae*	48.contig2_	Plasminog	2	19.1	8.76	86.3	8.98E+05	(R)FLCSG	6.63	5.66	1.00	646.86	8.75	7.58%	4.92%	0.37%
														3	17.99	17.99	88.2	2.05E+06	(K)HIAPLS	6.68	5.66	1.00	654.35	9.75			
														2	15.09	15.09	82	6.84E+05	(K)HIAPLS	6.68	5.66	1.00	981.02	9.75			
														3	17.2	8.98	76.1	2.70E+05	(K)WVITA	6.82	5.66	0.90	858.08	6.00			
														2	16.46	16.46	71.9	7.48E+05	(R)IMGW	7.52	5.66	1.00	613.82	8.75			
15	3	3.1	10	5	94.92	48.4	2.74E+07	2.74E+06	15775.9	9.55	*Tropidolae*	23.contig1	Basic phos	3	24.38	24.38	92.6	1.19E+06	(K)AIACGI	4.33	5.10	0.95	767.63	4.18	22.33%	4.92%	1.10%
														2	24.14	24.14	95.9	8.69E+04	(K)AIACGI	4.35	1.34	0.95	1150.94	4.18			
														2	12.32	12.32	81.7	3.05E+05	(K)mDHYS	5.45	5.66	0.96	737.33	6.49			
														3	12.79	12.79	77.2	2.96E+05	(K)mDHYS	5.45	5.66	0.95	491.89	6.49			
														3	14.33	14.33	84.7	1.16E+07	(K)MDHYS	5.70	16.72	0.97	486.56	6.49			
														2	13.41	13.41	92.4	8.82E+06	(K)MDHYS	5.70	16.72	1.00	729.33	6.49			
														2	15.59	6.23	88.9	1.14E+06	(K)TLAICL	5.97	5.53	0.99	438.26	8.41			
														3	16.95	7.07	92.1	2.96E+04	(K)TLAICL	6.52	0.13	0.89	575.31	8.17			
														2	23.67	23.67	91.9	2.54E+06	(K)NAFNY	6.80	0.13	0.98	1007.93	8.50			
														3	21.05	21.05	89.5	1.39E+06	(K)NAFNY	6.80	0.13	0.99	672.29	8.50			
15	3	3.2	8	3	53.84	43.6	2.51E+07	3.14E+06	12287	8.89	*Naja kaou*	60.contig2_	Phospholi	3	12.28	12.28	83.4	9.16E+04	(K)AIACGI	4.58	2.36	0.84	633.25	4.24	25.57%	4.92%	1.26%
														2	15.84	15.84	86.3	1.13E+05	(K)AIACGI	4.58	2.36	0.91	949.36	4.24			
														2	12.32	12.32	81.7	3.05E+05	(K)mDHYS	5.45	5.66	0.96	737.33	6.49			
														3	12.79	12.79	77.2	2.96E+05	(K)mDHYS	5.45	5.66	0.95	491.89	6.49			
														3	14.33	14.33	84.7	1.16E+07	(K)MDHYS	5.70	16.72	0.97	486.56	6.49			
														2	13.41	13.41	92.4	8.82E+06	(K)MDHYS	5.70	16.72	1.00	729.33	6.49			
														2	23.67	23.67	91.9	2.54E+06	(K)NAFNY	6.80	0.13	0.98	1007.93	8.50			
														3	21.05	21.05	89.5	1.39E+06	(K)NAFNY	6.80	0.13	0.99	672.29	8.50			
15	4	4.1	8	3	69.1	22.6	2.47E+07	3.09E+06	29040.6	7.93	*Tropidolae*	jene24832_	Beta-fibrin	3	15.54	15.54	75.7	7.69E+05	(R)VmGW	5.92	5.66	0.97	897.74	5.32	25.17%	4.92%	1.24%
														3	23	23	93	1.34E+07	(R)VMGW	6.32	11.19	0.88	892.41	5.32			
														2	25.78	25.78	95.1	1.27E+06	(R)VMGW	6.32	5.66	0.99	1338.12	5.32			
														4	15.36	15.36	71.1	1.09E+06	(R)VMGW	6.32	11.19	0.98	669.56	5.32			
														4	18.04	9.36	86.8	1.40E+05	(R)VMGW	7.65	5.67	0.89	917.18	5.38			
														3	21.47	21.47	96.1	6.90E+06	(K)GDSG	8.53	10.81	0.91	956.45	4.65			
														2	25.28	25.28	96	4.90E+05	(K)GDSG	8.53	5.40	0.99	1434.17	4.65			
														4	14.59	14.59	75.5	6.16E+05	(K)GDSG	8.53	10.81	0.96	717.59	4.65			
15	5	5.1	2	2	34.08	13.2	2.00E+05	1.00E+05	19250.4	6.51	*Tropidolae*	jene14693_	Galactose	2	16.13	16.13	81.2	1.45E+05	(K)DFSWI	7.27	0.13	0.99	621.27	4.03	0.82%	4.92%	0.04%
														2	17.95	17.95	86.1	5.56E+04	(K)GQAE	8.37	9.54	0.99	715.36	4.37			
								1.23E+07																	100.00%		
16	1	1.1	32	11	229.56	36.7	7.70E+07	2.41E+06	66461.8	6.24	*Tropidolae*	igene330_	Metallopro	2	25.23	25.23	93.2	2.33E+06	(K)LTPGS	4.82	5.40	0.95	1006.41	4.37	20.36%	2.79%	0.57%
														3	21.02	21.02	90.9	1.65E+06	(K)LTPGS	4.82	5.40	0.93	671.27	4.37			
														2	15.72	15.72	73.7	1.17E+06	(R)NDPDY	5.15	0.13	0.99	713.81	3.93			
														2	15.97	15.97	80.7	2.10E+06	(K)CPTDL	5.32	5.66	0.99	501.76	5.84			
														2	23.45	23.45	90.5	1.08E+07	(R)NDPDY	5.85	5.66	0.96	705.82	3.93			
														3	14.46	14.46	73.8	3.05E+05	(R)NDPDY	5.85	5.66	0.99	470.88	3.93			
														4	12.24	1.54	77.4	5.65E+06	(K)AEYQT	6.10	5.66	0.98	650.60	9.53			
														3	20.26	20.26	94.2	1.54E+06	(K)AEYQT	6.10	5.66	0.99	867.13	9.53			
														2	18.57	11.81	83	8.86E+05	(K)FCVIVI	6.27	0.13	0.99	1095.50	4.22			
														4	14.93	14.93	74	2.05E+06	(K)TDIISP	7.47	5.53	0.92	1108.45	3.66			
														2	22.47	22.47	88.7	7.25E+05	(R)VALVC	7.83	0.13	0.97	824.89	4.37			
														3	16.51	16.51	80.1	1.37E+05	(R)VALVC	7.83	0.13	0.95	550.27	4.37			
														3	19.16	19.16	96	4.52E+06	(R)RSPDN	7.97	11.06	1.00	1087.86	3.97			
														2	19.37	15.16	92.3	2.44E+05	(R)RSPDN	7.95	5.66	0.97	1631.29	3.97			
														3	18.68	18.68	92.1	5.01E+06	(R)SPDNA	8.52	55.32	1.00	1035.83	3.66			
														2	22.11	22.11	97.3	1.35E+06	(R)SPDNA	8.42	33.32	1.00	1553.24	3.66			
														4	15.61	7.23	87.1	2.90E+05	(K)CPImT	8.30	0.13	0.82	910.69	5.96			
														3	23.66	17.79	92	1.38E+06	(K)CPIMTI	8.55	5.66	0.87	1208.58	5.96			
														4	15.1	4.47	87	1.27E+06	(K)CPIMTI	8.55	5.66	0.92	906.69	5.96			
														3	15.24	11.4	87.5	2.51E+05	(R)IIYEIVN	8.73	55.32	1.00	766.06	4.53			
														3	23.54	23.54	90.6	5.80E+06	(R)IIYEIVN	10.12	19.47	0.99	760.73	4.53			
														2	22.85	18.74	98.3	1.48E+06	(R)IIYEIVN	10.10	16.60	0.99	1140.59	4.53			
														4	14.95	5.75	86.4	2.82E+04	(R)IIYEIVN	10.08	10.37	0.90	570.80	4.53			
														3	16.06	16.06	81.5	2.19E+06	(R)RSPDN	10.55	151.96	1.00	1087.86	3.97			
														3	18.33	18.33	90.9	3.68E+06	(R)SPDNA	10.63	145.63	1.00	1035.83	3.66			
														3	19.29	19.29	89.8	5.04E+05	(R)IIYEIVN	12.07	7.99	0.99	760.73	4.53			
														3	19.72	19.72	100	4.13E+06	(R)SPDNA	12.13	76.61	1.00	1035.83	3.66			
														3	13.03	13.03	73.4	6.59E+06	(R)SPDNA	13.78	117.91	0.95	1035.83	3.66			
														3	19.99	19.99	90.9	4.55E+06	(R)IIYEIVN	13.73	105.55	0.99	760.73	4.53			
														3	10.89	10.89	73.4	4.39E+06	(R)SPDNA	14.10	89.21	0.90	1035.83	3.66			
														3	10.29	10.29	71.7	7.87E+03	(R)RSPDN	13.63	0.00	0.87	1087.86	3.97			
														3	12.94	12.94	78.6	1.11E+04	(R)SPDNA	14.02	0.00	0.91	1035.83	3.66			
16	2	2.1	16	13	227.83	38.9	1.08E+07	6.75E+05	54759.7	6.3	*Tropidolae*	502.contig1	Intestinal-t	2	16.77	16.77	83.5	9.29E+05	(K)GNYG	5.62	0.13	0.99	625.83	8.75	5.71%	2.79%	0.16%

												2	21.22	13.43	94.9	1.29E+06	(R)ALTEA	6.17	0.13	0.99	568.80	4.14			
												2	19.28	19.28	77	5.90E+05	(R)QVPDS	6.25	5.66	0.99	869.43	5.83			
												2	17.37	17.37	85.1	3.33E+05	(K)AYTSIV	6.35	0.13	1.00	973.98	5.88			
												3	17.69	5.55	87.2	1.98E+05	(K)AYTSIV	6.35	0.13	0.99	649.66	5.88			
												2	14.55	8.39	80.9	1.15E+06	(R)GASIF	6.82	0.13	1.00	509.29	8.75			
												2	22.26	17.66	94	3.52E+05	(R)NWYS	6.83	5.66	1.00	991.95	4.37			
												3	20.66	20.66	88.7	1.77E+05	(R)NWYS	6.83	5.66	1.00	661.64	4.37			
												2	16.29	8.31	88.1	1.61E+06	(R)IDLLQ	7.18	5.54	1.00	599.84	4.21			
												2	14.33	14.33	71.5	4.97E+05	(R)LMGLF	7.47	5.66	0.98	634.31	4.37			
												4	11.53	2.65	74.9	3.21E+05	(R)EEDTL	7.58	5.66	0.98	664.07	4.72			
												2	16.34	16.34	85.5	4.06E+05	(K)GFYLF	7.67	5.66	1.00	572.79	6.00			
												3	21.92	21.92	100	4.71E+05	(K)GHMEI	8.02	0.13	0.98	906.43	4.65			
												2	15.37	6.12	75.7	1.82E+06	(R)NLIEEV	8.30	0.13	1.00	536.79	4.53			
												2	20.28	14.78	94.1	3.58E+05	(K)NLILFL	9.58	0.13	0.99	995.05	5.84			
												3	16.7	6.08	93.8	2.65E+05	(K)NLILFL	9.58	0.13	0.99	663.70	5.84			
16	3	3.1	16	9	176.21	36	1.69E+07	1.06E+06	36683.3	9.32	*Tropidolae* gene24659_ Snake ven	2	14.19	14.19	89	6.92E+05	(K)VGIIGY	5.68	5.66	1.00	476.28	8.56	8.94%	2.79%	0.25%
												3	19.82	15.82	92.8	4.72E+06	(K)IIALGH	6.47	5.66	1.00	519.28	5.32			
												2	26.43	22.88	98.7	7.67E+05	(K)IIALGH	6.47	5.66	0.99	778.42	5.32			
												2	15.03	10.08	71.2	3.04E+06	(R)QVPVV	6.60	11.19	0.97	653.86	8.59			
												3	10.57	4.5	72.3	8.01E+04	(R)QVPVV	6.58	5.66	0.97	436.24	8.59			
												2	21.25	16.91	96.2	1.60E+06	(K)ETPVL	7.55	5.66	0.99	859.94	4.53			
												3	18.86	14.09	86.9	3.65E+05	(K)ETPVL	7.55	5.66	0.99	573.63	4.53			
												3	15.76	10.22	89.4	3.01E+06	(K)ETPVL	8.15	5.66	0.99	897.12	4.14			
												2	20.91	20.91	97.2	2.96E+05	(K)ETPVL	8.15	5.66	1.00	1345.17	4.14			
												3	17.91	8.64	90	3.41E+05	(K)YLGYL	8.92	11.06	1.00	652.70	5.96			
												2	20.53	15.54	91.6	6.88E+05	(K)YLGYL	9.23	5.66	1.00	722.89	4.21			
												3	15.52	7.16	85.1	4.53E+04	(K)YLGYL	9.23	5.66	1.00	482.26	4.21			
												3	19.27	15.09	97.6	6.96E+05	(R)YDAM	9.40	0.13	0.81	858.43	4.22			
												3	18.39	11	85.1	4.62E+05	(R)HVLLL	9.50	42.61	1.00	834.42	5.21			
												2	21.57	14.72	96.5	8.63E+04	(R)YDAM	9.40	0.13	0.86	1287.14	4.22			
												4	14.79	8.19	86.8	2.11E+04	(R)YDAM	9.40	0.00	0.95	644.07	4.22			
16	3	3.2	15	7	138.86	41.7	1.66E+07	1.11E+06	29358.2	7.3	*Tropidolae* gene24655_ Snake ven	3	13.25	13.25	79	3.25E+05	(K)IQLHN	4.97	0.13	0.98	506.26	6.75	9.36%	2.79%	0.26%
												3	12.74	12.74	78.3	3.14E+05	(R)HGQG	5.80	0.13	0.98	523.94	6.75			
												2	11.96	11.96	88	2.56E+05	(R)HGQG	5.80	0.13	1.00	785.41	6.75			
												3	17	17	79.1	8.02E+04	(R)HTDDI	6.08	0.00	0.37	809.04	6.00			
												2	20.06	20.06	100	3.11E+06	(R)VVSLN	6.60	5.66	0.99	724.87	9.72			
												3	18.81	18.81	93.9	3.21E+05	(R)VVSLN	6.60	5.66	0.96	483.58	9.72			
												2	21.4	17.68	100	3.34E+06	(R)VPAYV	6.72	5.66	0.98	573.82	5.97			
												3	15.5	15.5	88.5	3.15E+05	(R)HGQG	6.82	5.53	1.00	518.61	6.75			
												2	20.51	20.51	93	1.71E+05	(R)HGQG	6.83	5.66	1.00	777.41	6.75			
												3	23.56	23.56	100	2.31E+06	(R)FHECN	8.02	0.13	1.00	803.38	4.54			
												2	23.24	23.24	93.4	4.73E+05	(R)FHECN	8.02	0.13	0.99	1204.56	4.54			
												4	12.93	12.93	74.7	1.47E+05	(R)FHECN	8.02	5.53	0.95	602.78	4.54			
												3	13.32	5.71	83	3.59E+05	(K)LLLPSI	8.12	5.66	1.00	645.67	6.74			
												3	19.77	8.85	95.1	4.73E+06	(K)LLLPSI	8.65	5.53	0.98	640.34	6.74			
												2	23.08	16.15	98.2	3.33E+05	(K)LLLPSI	8.65	11.06	0.99	960.01	6.74			
16	4	4.1	16	7	130.78	41	3.93E+07	2.46E+06	27338.7	9.3	*Tropidolae* gene19683_ GPX3	2	19.38	12.09	86.1	6.06E+06	(K)QEPG	5.80	0.13	0.97	770.90	4.53	20.78%	2.79%	0.58%
												3	12.37	12.37	77.1	8.77E+05	(K)QEPG	5.80	0.13	0.97	514.27	4.53			
												2	21.84	21.84	85.4	6.15E+06	(K)NSCPF	5.92	5.66	0.99	802.38	4.37			
												3	17.86	17.86	92.5	3.18E+05	(K)NSCPF	5.92	5.66	0.99	535.25	4.37			
												2	12.47	4.32	88.8	5.04E+06	(R)IYTFLK	6.28	5.66	0.95	392.74	8.59			
												2	12.01	12.01	70.6	3.39E+05	(K)LFWSF	6.35	5.53	1.00	462.74	8.75			
												2	17.8	7.63	88.2	3.22E+06	(K)LFWSF	7.10	5.66	0.97	454.74	8.75			
												3	14.53	14.53	95.4	3.17E+06	(K)HVRPG	7.37	0.13	1.00	643.35	11.00			
												2	16.45	4.36	89.3	2.07E+05	(K)HVRPG	7.37	0.13	0.99	964.52	11.00			
												3	24.51	24.51	97	3.75E+06	(K)VDCYD	8.23	44.38	1.00	1007.46	4.22			
												4	16.12	16.12	86.9	9.03E+05	(K)VDCYD	8.37	66.38	0.99	755.85	4.22			
												2	18.33	11.93	93.1	5.65E+06	(R)LVILGF	8.10	0.13	0.91	746.90	8.75			
												2	22.77	22.77	82.1	2.93E+05	(K)VDCYD	8.17	38.72	0.96	1510.69	4.22			
												3	16.8	16.8	89.4	5.85E+05	(R)LVILGF	8.10	0.13	0.99	498.27	8.75			
												3	20.18	20.18	78.3	2.16E+06	(K)VDCYD	10.48	150.67	1.00	1007.46	4.22			
												3	21.42	21.42	84.1	5.60E+05	(K)VDCYD	11.47	30.59	0.99	1007.46	4.22			
16	5	5.1	10	5	99.33	38.6	1.51E+07	1.51E+06	19250.4	6.51	*Tropidolae* gene14693_ Galactose	3	16.32	16.32	95	4.19E+06	(R)SAESH	5.98	0.13	1.00	626.96	4.72	12.77%	2.79%	0.36%
												2	22.2	22.2	95.6	1.08E+06	(R)SAESH	5.98	0.13	0.98	939.93	4.72			
												3	15.6	15.6	85.1	4.86E+04	(K)KDFSW	6.53	0.13	0.99	457.21	4.56			
												2	19.96	19.96	95.6	3.59E+04	(K)KDFSW	6.53	0.13	0.99	685.31	4.56			
												2	18.4	12.55	92	4.12E+06	(K)DFSWI	7.28	5.66	0.98	621.27	4.03			
												2	19.55	19.55	91	4.34E+06	(K)GQAEV	8.35	11.19	1.00	715.37	4.37			
												3	10.03	10.03	71.3	2.76E+05	(K)GQAEV	8.35	11.19	0.99	477.25	4.37			
												3	19.22	19.22	100	7.37E+05	(K)EFCVE	9.47	87.66	0.96	921.76	4.00			
												2	16.68	16.68	80.3	8.10E+04	(K)EFCVE	9.13	16.73	0.94	1382.13	4.00			
												2	13.91	13.91	94.2	2.22E+05	(K)GQAEV	10.12	103.36	0.89	715.36	4.37			
16	6	6.1	8	5	94.74	31.3	2.39E+06	2.99E+05	28709.3	6.09	*Tropidolae* 48.contig1_ Serine pro	2	20.52	20.52	79.1	6.57E+05	(K)DTCVG	5.35	0.13	0.98	810.86	4.21	2.53%	2.79%	0.07%
												3	18.6	18.6	85.7	1.65E+05	(K)DTCVG	5.35	0.13	0.99	540.91	4.21			
												4	14.48	9.13	82.7	3.25E+05	(K)VKLPD	7.65	0.13	1.00	557.05	5.30			
												3	13.9	13.9	75.4	2.61E+05	(K)LPDVP	7.73	5.66	0.99	666.67	4.41			
												3	23.05	23.05	97	4.20E+05	(R)EFQGI	8.58	44.25	0.99	924.10	4.26			
												2	22.21	22.21	90.8	1.78E+05	(R)EFQGI	8.50	22.25	0.99	1385.64	4.26			
												3	17.66	17.66	81.7	2.02E+05	(R)AAYPD	8.67	5.53	0.99	746.04	4.14			
												2	22.79	22.79	82.4	1.83E+05	(R)AAYPD	8.65	5.53	0.99	1118.56	4.14			
16	7	7.1	6	6	90.51	15.6	1.19E+06	1.98E+05	58913.9	8.52	*Tropidolae* gene22180_ L-amino a	2	12.6	6.61	74.2	2.89E+05	(K)EVTVT	5.43	0.13	0.98	605.82	6.10	1.68%	2.79%	0.05%
												2	14.4	14.4	83.8	1.79E+05	(K)NAWYI	6.68	5.66	1.00	464.24	8.59			
												2	15.28	15.28	70.2	1.92E+05	(R)ETDYE	7.77	5.66	0.99	757.86	3.91			
												3	12.92	1.32	74.8	1.03E+05	(R)KFGLG	7.83	0.13	0.99	581.30	6.14			
												2	19.89	13.9	88.7	1.90E+05	(K)FGLQL	8.57	0.13	0.99	807.40	4.53			
												4	15.42	15.42	78.1	2.33E+05	(K)HVVIV	10.28	9.93	0.97	908.74	5.32			
16	8	8.1	5	5	90.46	13.1	9.00E+05	1.80E+05	50974.4	9.34	*Tropidolae* 504.contig1 Zinc metal	2	20.96	20.96	90.2	2.85E+05	(K)ASYLF	5.72	0.13	0.99	603.27	5.88	1.52%	2.79%	0.04%
												2	11.7	11.7	88.9	1.56E+05	(K)LFAAW	6.28	5.66	1.00	382.22	9.75			
												2	19.96	19.96	79.7	2.64E+05	(R)YSPYD	6.45	5.66	0.99	886.91	4.03			
												2	19.11	10.7	93.3	1.63E+05	(K)LYLVAI	8.42	5.66	0.97	735.41	5.83			
												3	18.73	18.73	87.2	3.21E+04	(R)IYNMA	10.53	5.22	0.97	577.98	8.75			
16	9	9.1	6	4	77.62	54.4	2.74E+06	4.57E+05	18686.1	9.4	*Tropidolae* nigene18_T Agglucetin	3	18.11	18.11	86.5	1.58E+05	(R)IINKPQ	4.80	0.00	0.99	500.60	6.07	3.86%	2.79%	0.11%
												3	17.98	17.98	71.3	1.66E+06	(K)CPSEV	5.58	5.66	1.00	645.59	5.32			
												3	20.92	20.92	87	2.47E+05	(K)QCSTE	9.00	16.73	0.99	914.07	3.92			
												4	16.95	8.54	88.7	5.74E+05	(K)GGHLV	10.92	6.56	1.00	1012.01	5.28			
												5	14.3	3.34	88.5	6.53E+04	(K)GGHLV	10.92	6.56	0.93	809.81	5.28			
												3	20.61	20.61	87.4	3.60E+04	(K)GGHLV	10.92	6.56	0.87	1349.00	5.28			
16	10	10.1	4	3	55.96	36.9	5.09E+06	1.27E+06	16330.1	6.31	*Tropidolae* gene23090_ C-type lect	3	12.73	5.01	89.4	9.14E+05	(K)GSHLV	6.02	72.03	0.98	656.67	5.28	10.77%	2.79%	0.30%
												2	21.26	21.26	92.1	1.71E+06	(R)WSSYI	5.72	0.13	1.00	845.87	6.75			
												2	21.97	21.97	96.1	1.01E+06	(R)TFDNG	7.83	5.53	0.97	1205.51	4.21			
												4	10.71	4.08	78.5	1.46E+06	(K)GSHLV	8.98	218.15	0.99	492.75	5.28			
16	11	11.1	2	2	38.97	20.7	1.49E+05	7.45E+04	15775.9	9.55	*Tropidolae* 723.contig1 Basic phos	3	23.01	23.01	95.3	9.86E+04	(K)AIACGI	4.33	3.19	0.96	767.63	4.18	0.63%	2.79%	0.02%
												2	15.96	6.15	92.7	5.03E+04	(K)TLAICL	6.03	11.19	0.99	438.25	8.41			
16	12	12.1	2	2	37.76	8.9	2.58E+05	1.29E+05	35547.1	8.56	*Tropidolae* 565.contig1 ADP-ribos	2	20.25	20.25	88.1	1.78E+05	(R)VTEED	7.20	11.19	0.98	840.39	3.91	1.09%	2.79%	0.03%
												3	17.51	17.51	85.9	8.03E+04	(R)GTTEH	8.02	0.13	0.98	479.94	5.40			
								1.18E+07															100.00%		
17	1	1.1	30	16	296.03	41.4	8.76E+07	2.92E+06	58913.9	8.52	*Tropidolae* gene22180_ L-amino a	3	13.74	3.91	88.5	1.39E+06	(K)RVWE	5.17	0.13	1.00	513.96	8.59	19.42%	7.61%	1.48%
												2	18.17	14.92	91.1	5.19E+05	(K)SAGQL	5.23	5.66	1.00	641.32	4.53			
												2	10.5	5.76	72	5.39E+06	(K)EVTVT	5.35	22.25	0.98	605.82	6.10			
												2	19.17	11.1	94.5	1.89E+06	(R)VWEVI	5.53	0.13	0.99	692.39	6.04			
												2	14.64	14.64	85.4	3.74E+06	(K)FEPPL	5.73	44.25	0.99	462.76	6.00			
												3	18.09	18.09	95.5	3.54E+06	(K)KDIQAI	6.23	5.66	1.00	566.30	8.50			
												2	17.53	11.6	90.4	1.55E+06	(K)KDIQAI	6.23	5.66	1.00	848.94	8.50			
												2	18.3	18.3	81.6	5.10E+06	(K)ETSSV	6.82	55.31	0.99	929.95	4.37			
												3	18.16	13.45	80.4	1.71E+06	(K)ETSSV	6.78	38.85	0.99	620.30	4.37			
												2	16.5	16.5	85.7	7.41E+05	(K)EGWY	6.80	5.66	1.00	647.31	6.10			
												2	19.29	10.76	91.7	1.39E+06	(K)DIQAF	6.95	5.66	1.00	784.89	5.83			
												3	12.62	12.62	76.9	1.95E+05	(K)DIQAF	6.95	5.66	0.99	523.60	5.83			
												3	19.95	12.46	93	4.57E+06	(R)IYFAGI	7.47	11.19	1.00	757.71	5.32			
												2	28.5	28.5	100	1.78E+06	(R)IYFAGI	7.92	99.57	0.85	1136.06	5.32			
												3	13.7	1.65	78.8	4.98E+06	(R)KFGLG	7.73	16.72	1.00	581.30	6.14			

														2	19.94	14.13	93.8	2.90E+06	(R)KFGLC	7.73	16.72	1.00	871.45	6.14			
														3	14.08	14.08	78.9	1.04E+06	(R)ETDYE	7.67	5.66	0.98	505.57	3.91			
														3	13.22	7	70.1	1.57E+06	(K)EVTVT	7.78	203.64	0.97	605.82	6.10			
														2	17.74	12.48	86.1	3.18E+06	(K)FGLQL	8.48	0.13	1.00	807.40	4.53			
														2	19.69	19.69	89	2.72E+06	(K)DCADI	8.68	5.66	1.00	1049.54	4.41			
														3	20.18	20.18	79.4	5.64E+05	(K)YAMG	8.92	5.66	0.96	1022.16	6.75			
														2	12.47	12.47	100	1.57E+06	(K)ETSSV	8.92	173.54	0.99	929.95	4.37			
														3	21.21	13.95	93.3	8.73E+06	(R)IYFAGI	10.40	184.92	1.00	757.71	5.32			
														4	17.88	17.88	91.5	4.52E+05	(K)HVVIV	9.70	5.66	0.96	912.74	5.32			
														3	17.41	17.41	76.4	9.30E+04	(K)HVVIV	9.70	5.66	0.91	1216.64	5.32			
														4	16.64	16.64	78.3	3.22E+06	(K)HVVIV	10.25	9.03	1.00	908.74	5.32			
														5	15.76	15.76	74.4	8.76E+05	(K)HVVIV	10.25	9.03	1.00	727.19	5.32			
														3	24.78	20.82	90.8	5.76E+05	(K)HVVIV	10.25	5.40	0.96	1211.31	5.32			
														3	18.85	12.2	91.1	9.54E+06	(R)IYFAGI	11.83	122.37	1.00	757.71	5.32			
														3	18.08	10.09	86.7	1.21E+07	(R)IYFAGI	13.90	158.96	0.99	757.71	5.32			
17	2	2.1	13	10	181.25	25.1	7.20E+06	5.54E+05	72248.1	8.91	*Tropidolae*	548.contig1	Phosphodi	2	18.76	18.76	90.2	1.33E+06	(K)AATYF	6.87	5.66	0.99	678.34	6.04	3.68%	7.61%	0.28%
														2	15.2	15.2	72.8	1.82E+05	(K)SMQAI	7.02	11.19	0.96	752.39	8.49			
														2	16.33	16.33	81.4	1.15E+06	(K)DFYTF	7.12	5.66	1.00	710.84	4.03			
														2	15.55	9.29	85.6	2.32E+05	(R)TLGML	7.43	16.72	0.99	546.79	5.66			
														4	11.65	1.51	85	9.51E+05	(K)RPDFY	7.45	5.66	1.00	803.65	4.50			
														3	16.41	16.41	79	6.85E+05	(K)NPAW	7.68	11.19	0.99	771.05	8.60			
														2	22.97	22.97	91.1	2.29E+05	(K)NPAW	7.67	11.19	1.00	1156.07	8.60			
														2	21.96	16.89	97	1.99E+06	(R)VTEVL	8.70	5.66	1.00	707.40	4.37			
														3	14.39	14.39	78.1	1.04E+05	(R)VTEVL	8.70	5.66	0.99	471.94	4.37			
														3	21.06	21.06	92.5	1.04E+05	(K)CASSI	10.05	5.53	0.89	997.80	4.14			
														3	19.4	19.4	92.7	1.06E+05	(K)EKNEV	10.10	5.66	0.97	895.44	4.25			
														3	15.53	8.85	89.1	8.85E+04	(K)NEVPS	10.52	5.22	0.94	809.72	4.00			
														2	18.37	12.58	88.9	4.68E+04	(K)NEVPS	10.52	5.22	0.97	1214.08	4.00			
17	2	2.2	9	7	118.85	13.5	6.46E+06	7.18E+05	98254	8.74	*Crotalus a*	J3SEZ3	Venom ph	4	10.98	4.46	79.5	5.44E+04	(K)TESEA	4.90	5.40	0.93	602.06	6.62	4.77%	7.61%	0.36%
														2	18.76	18.76	90.2	1.33E+06	(K)AATYF	6.87	5.66	0.99	678.34	6.04			
														2	16.33	16.33	81.4	1.15E+06	(K)DFYTF	7.12	5.66	1.00	710.84	4.03			
														2	15.55	9.29	85.6	2.32E+05	(R)TLGML	7.43	16.72	0.99	546.79	5.66			
														3	16.41	16.41	79	6.85E+05	(R)NPAW	7.68	11.19	0.99	771.05	8.60			
														2	22.97	22.97	91.1	2.29E+05	(R)NPAW	7.67	11.19	1.00	1156.07	8.60			
														3	19.17	19.17	92.2	6.41E+05	(K)IPIPTH	9.03	0.13	0.95	1113.58	6.75			
														4	19.34	8.29	93.6	5.55E+05	(K)IPIPTH	9.03	0.13	0.96	835.44	6.75			
														2	14.92	9.83	93.3	1.58E+06	(K)TFLPIF	9.33	5.66	1.00	630.85	5.18			
17	2	2.3	7	5	87.7	35.1	3.26E+06	4.66E+05	26848	6.32	*Tropidolae*	gene21602_	Phosphodi	2	16.69	16.69	75.4	6.02E+04	(R)SQPTS	4.62	11.06	0.98	787.37	5.55	3.10%	7.61%	0.24%
														3	19.17	19.17	92.2	6.41E+05	(K)IPIPTH	9.03	0.13	0.95	1113.58	6.75			
														4	19.34	8.29	93.6	5.55E+05	(K)IPIPTH	9.03	0.13	0.96	835.44	6.75			
														3	18.13	18.13	94	1.95E+05	(R)VRDVE	9.15	5.66	0.99	1024.56	4.68			
														2	14.92	9.83	93.3	1.58E+06	(K)TFLPIF	9.33	5.66	1.00	630.85	5.18			
														3	18.62	12.02	100	1.89E+05	(R)DVELL	9.70	5.66	0.98	939.51	4.14			
														2	15.51	15.51	83.2	4.33E+04	(R)DVELL	9.70	5.66	0.89	1408.75	4.14			
17	3	3.1	15	9	178.92	26.1	2.45E+07	1.63E+06	50974.4	9.34	*Tropidolae*	504.contig1	Zinc metal	3	21.53	21.53	94.8	3.65E+05	(R)LYCVA	4.98	0.13	1.00	655.32	9.99	10.86%	7.61%	0.83%
														2	18.3	13.28	91.1	1.03E+07	(K)IIVQSS	5.75	5.66	0.90	650.38	5.84			
														3	11.82	3.92	72.7	5.04E+05	(K)IIVQSS	5.75	5.66	1.00	433.92	5.84			
														2	13.27	13.27	84.1	1.02E+06	(K)LFAAW	6.27	5.53	1.00	382.22	9.75			
														2	24.89	20.25	98.2	8.71E+05	(R)YSPYD	6.37	0.13	1.00	886.91	4.03			
														2	14.34	6.55	77.3	3.17E+04	(K)LYLVAI	7.70	5.66	0.98	743.40	5.83			
														3	19.94	19.94	75.1	1.65E+06	(R)DQCIS	8.17	33.19	0.96	893.73	4.21			
														2	25.95	25.95	87.7	1.37E+06	(R)DQCIS	8.15	27.79	0.97	1340.09	4.21			
														3	18.7	18.7	94.5	3.27E+05	(R)MNIHV	8.22	5.66	0.93	576.31	8.52			
														2	20.22	11.35	89.3	3.96E+06	(K)LYLVAI	8.33	5.66	0.99	735.41	5.83			
														3	10.13	10.13	78.8	1.13E+05	(K)IIVQSS	8.72	11.19	0.99	682.06	8.75			
														3	19.17	19.17	93.3	2.21E+05	(R)IYNmA	9.22	0.13	0.98	583.32	8.75			
														3	18.81	18.81	82	1.43E+06	(R)DQCIS	10.70	148.99	0.98	893.73	4.21			
														3	20.21	20.21	95.6	2.00E+06	(R)IYNMA	10.52	5.22	1.00	577.99	8.75			
														2	25.93	25.93	98.5	3.54E+05	(R)IYNMA	10.52	5.22	0.99	866.47	8.75			
17	4	4.1	9	6	122.85	36.4	8.37E+06	9.30E+05	30421.9	5.14	*Tropidolae*	gene22208_	Gastric int	2	14.33	0.91	85.8	1.92E+06	(K)EALILI	6.45	0.13	1.00	450.79	6.10	6.19%	7.61%	0.47%
														2	21.74	14.7	95.6	1.64E+06	(K)LIGPYI	7.20	0.13	1.00	759.40	6.00			
														2	20.84	20.84	87.7	5.15E+05	(K)TYLDV	7.97	66.51	0.97	1142.05	4.37			
														3	17.45	17.45	96.2	1.09E+06	(K)IPEGAI	8.48	0.13	0.99	718.73	6.00			
														2	20.45	14.81	95	1.60E+06	(K)IPEGAI	8.53	5.66	0.99	1077.59	6.00			
														3	21.24	21.24	95.3	1.21E+06	(R)LSVDT	8.75	38.84	0.99	706.03	5.32			
														3	18.72	9.89	92.3	6.68E+04	(K)TTYYC	9.87	0.13	0.99	672.68	4.37			
														2	24.25	24.25	100	4.65E+04	(K)TTYYC	9.87	0.13	0.98	1008.52	4.37			
														3	10.65	10.65	72.5	2.80E+05	(R)LSVDT	10.03	92.49	0.92	706.03	5.32			
17	5	5.1	6	6	105.95	4.6	1.43E+06	2.38E+05	185878.2	5.98	*Naja suma*	gene370_N	Cobra ven	2	17.96	17.96	83.9	2.63E+05	(R)INYEN	6.28	5.66	0.99	588.82	6.00	1.59%	7.61%	0.12%
														2	17.68	12.71	86.8	3.64E+05	(K)GIYTP	6.37	0.13	1.00	661.86	8.59			
														2	16.25	8.46	95.5	2.66E+05	(R)IDVPL	6.45	0.13	1.00	527.81	4.37			
														2	20.11	20.11	91	2.31E+05	(K)VNDDY	6.97	11.19	0.95	669.33	4.21			
														3	16.09	16.09	74.9	2.87E+05	(K)QLDIF	7.28	0.13	0.99	462.91	5.21			
														3	17.86	10.95	87.2	2.30E+04	(R)AVPFV	9.97	0.13	0.69	825.79	4.65			
17	5	5.2	7	6	101.36	5	1.46E+06	2.09E+05	175754.7	6.35	*Naja suma*	60.contig1_	Cobra ven	2	17.96	17.96	83.9	2.63E+05	(K)INYEN	6.28	5.66	0.99	588.82	6.00	1.39%	7.61%	0.11%
														2	17.68	12.71	86.8	3.64E+05	(K)GIYTP	6.37	0.13	1.00	661.86	8.59			
														2	16.25	8.46	95.5	2.66E+05	(R)IDVPL	6.45	0.13	1.00	527.81	4.37			
														3	16.09	16.09	74.9	2.87E+05	(K)QLDIF	7.28	0.13	0.99	462.91	5.21			
														2	16.32	10.13	76.7	2.02E+05	(K)LILNTF	8.22	0.13	0.98	932.06	9.75			
														3	17.06	10.58	95	2.79E+04	(R)AVPFV	9.32	0.13	0.94	701.73	4.65			
														2	12.1	12.1	86.6	5.07E+04	(R)AVPFV	9.23	5.66	0.89	1052.09	4.65			
17	5	5.3	3	3	51.86	5.4	9.78E+05	3.26E+05	69677.2	7.38	*Naja kaou*	Q91132	Cobra ven	2	17.68	12.71	86.8	3.64E+05	(K)GIYTP	6.37	0.13	1.00	661.86	8.59	2.17%	7.61%	0.16%
														3	16.09	16.09	74.9	2.87E+05	(K)QLDIF	7.28	0.13	0.99	462.91	5.21			
														2	18.09	18.09	94.8	3.26E+05	(-)ALYTLI	8.28	5.66	1.00	665.91	8.79			
17	6	6.1	4	4	81.62	28.6	4.57E+06	1.14E+06	27338.7	9.3	*Tropidolae*	gene19683_	GPX3	2	18.27	10.67	85.7	1.23E+06	(K)QEPG	5.78	16.72	0.87	770.90	4.53	7.60%	7.61%	0.58%
														2	22.02	22.02	86.8	1.55E+06	(K)NSCPF	5.92	5.66	0.99	802.38	4.37			
														3	23.76	23.76	93.5	7.30E+05	(K)VDCYD	8.22	60.97	0.89	1007.46	4.22			
														2	17.57	11.24	92.5	1.06E+06	(R)LVILGF	8.05	5.66	1.00	746.90	8.75			
17	7	7.1	5	4	74.82	60	5.09E+06	1.02E+06	11260.7	6.11	*Tropidolae*	gene22208_	Gastric int	2	14.9	7.71	89.7	1.87E+06	(K)HVNLV	6.62	11.19	0.98	654.37	5.40	6.77%	7.61%	0.52%
														2	18.45	11.24	100	5.43E+05	(R)LAEDH	7.33	5.66	0.99	840.47	5.32			
														2	20.72	20.72	100	1.33E+06	(K)VALYV	7.88	5.66	1.00	509.33	8.72			
														3	20.75	20.75	86.4	7.98E+05	(R)FLQTC	8.93	5.66	0.99	965.86	6.07			
														4	17.55	17.55	81.1	5.47E+05	(R)FLQTC	8.93	5.66	0.98	724.65	6.07			
17	8	8.1	4	3	63.36	19.1	2.69E+06	6.73E+05	29358.2	7.3	*Tropidolae*	gene24655_	Snake ven	2	20.31	20.31	100	1.41E+06	(R)VVSLN	6.55	0.13	0.99	724.87	9.72	4.47%	7.61%	0.34%
														3	22.92	22.92	98.2	8.27E+05	(R)FHECN	7.93	0.13	0.99	803.38	4.54			
														2	25.58	25.58	95.4	2.27E+05	(R)FHECN	7.93	0.13	0.97	1204.56	4.54			
														3	17.47	7.38	93.7	2.29E+05	(K)LLLPSI	8.50	5.66	0.98	640.34	6.74			
17	8	8.2	3	3	51.75	6.9	1.79E+06	5.97E+05	65028.4	7.85	*Calloselas*	ene10279_	Snake ven	2	20.66	15.51	94.5	5.62E+05	(K)ETPVL	7.47	0.13	1.00	859.94	4.53	3.97%	7.61%	0.30%
														3	13.62	8.57	83.5	9.95E+05	(K)ETPVL	8.03	5.66	0.99	897.12	4.14			
														3	17.47	7.38	93.7	2.29E+05	(K)LLLPSI	8.50	5.66	0.98	640.34	6.74			
17	9	9.1	3	3	54.97	10.5	1.74E+06	5.80E+05	36683.3	9.32	*Tropidolae*	gene24659_	Snake ven	2	20.66	15.51	94.5	5.62E+05	(K)ETPVL	7.47	0.13	1.00	859.94	4.53	3.86%	7.61%	0.29%
														3	13.62	8.57	83.5	9.95E+05	(K)ETPVL	8.03	5.66	0.99	897.12	4.14			
														2	20.69	16.14	91	1.82E+05	(K)YLGYL	9.18	16.59	1.00	722.89	4.21			
17	10	10.1	10	3	59.77	36.9	1.77E+07	1.77E+06	16330.1	6.31	*Tropidolae*	gene23090_	C-type lect	4	10.36	3.16	79.5	2.36E+06	(K)GSHLV	5.82	60.85	0.99	492.75	5.28	11.77%	7.61%	0.90%
														3	11.78	4.51	93.6	3.53E+06	(K)GSHLV	6.67	166.07	1.00	656.67	5.28			
														2	15.62	15.62	89.6	6.02E+05	(K)GSHLV	5.83	60.98	0.99	984.50	5.28			
														2	20.28	20.28	88.7	3.13E+06	(R)WSSYI	5.63	0.13	1.00	845.87	6.75			
														2	19.39	19.39	90.1	3.57E+05	(R)TFDNC	7.20	0.13	0.97	1213.51	4.21			
														3	16.79	16.79	82.3	2.91E+05	(R)TFDNC	7.20	0.13	0.99	809.34	4.21			
														2	23.87	23.87	98.1	1.28E+06	(R)TFDNC	7.75	0.13	0.98	1205.51	4.21			
														4	10.72	10.72	85.1	2.95E+06	(K)GSHLV	8.90	219.53	0.99	492.75	5.28			
														4	11.36	11.36	82.2	1.76E+06	(K)GSHLV	10.83	128.90	0.98	492.75	5.28			
														3	10.23	10.23	78.6	1.40E+06	(K)GSHLV	11.08	110.27	0.98	656.67	5.28			
17	11	11.1	5	2	49.44	31.6	2.63E+06	5.26E+05	18686.1	9.4	*Tropidolae*	igene18_T	Agglucetin	3	20.63	20.63	80	1.72E+06	(K)CPSEV	5.42	5.66	0.99	645.59	5.32	3.50%	7.61%	0.27%
														2	25.02	25.02	98.1	4.24E+05	(K)CPSEV	5.42	5.66	0.99	967.88	5.32			
														4	20.63	20.63	96.7	4.06E+05	(K)GGHL	10.92	7.00	0.99	1012.01	5.28			
														3	24.42	24.42	94.9	2.78E+04	(K)GGHL	10.92	3.12	0.87	1349.00	5.28			
														5	13.2	3.65	83.8	4.68E+04	(K)GGHL	10.92	7.00	0.93	809.81	5.28			
17	12	12.1	2	2	37.83	4.7	1.05E+06	5.25E+05	64390.3	8.87	*Crotalus a*	F8S101	Phospholi	2	21.01	14.29	94.4	5.46E+05	(K)VVPES	7.92	11.19	1.00	666.85	4.53	3.49%	7.61%	0.27%

														2	16.82	11.19	85.2	5.04E+05	(R)SLEDG	8.12	0.13	0.99	852.96	4.14			
17	13	13.1	3	2	32.05	5.5	6.35E+05	2.12E+05	53091.1	9.42	*Ovophis o*	U3TBU1	Hyaluronid	2	15.52	15.52	84.4	3.81E+05	(R)NNQLL	8.57	5.66	1.00	621.84	9.75	1.41%	7.61%	0.11%
														2	16.53	10.67	88.7	1.80E+05	(R)DSTAL	10.05	10.05	1.00	933.50	4.37			
														3	12.19	12.19	73.3	7.44E+04	(R)DSTAL	10.05	10.17	0.98	622.67	4.37			
								1.50E+07																	100.00%		
18	1	1.1	13	11	169.03	8.7	1.38E+06	1.06E+05	175754.7	6.35	*Naja suma*	60.contig1_	Cobra ven	2	15.82	8.56	87	3.44E+04	(K)DLTEE	4.67	5.40	0.88	752.86	4.14	1.47%	2.57%	0.04%
														3	12.51	12.51	91.7	1.58E+05	(K)HFEVG	6.78	0.13	0.99	482.26	6.75			
														2	12.37	12.37	70.7	1.17E+05	(R)IPIIDGI	5.70	11.19	1.00	464.26	4.21			
														2	16.4	11.47	85.8	2.53E+05	(K)GIYTP(	6.60	5.53	1.00	661.86	8.59			
														2	10.5	6.03	82.8	3.08E+05	(R)IDVPL(	6.70	0.13	1.00	527.81	4.37			
														3	15.61	15.61	73.9	6.99E+04	(K)QLDIF\	7.53	5.40	0.99	462.91	5.21			
														2	17.28	17.28	88.7	7.06E+04	(K)YFTYL	7.80	5.40	0.97	587.83	8.50			
														3	18.2	11.56	87.7	2.93E+04	(K)LILNTF	8.40	0.00	0.94	621.71	9.75			
														2	18.12	12.49	83.6	1.84E+04	(K)LILNTF	8.40	0.00	0.95	932.06	9.75			
														2	22.45	22.45	92.7	9.80E+04	(R)SDFPE	9.13	12.49	1.00	754.87	4.37			
														2	12.19	7.51	91.2	2.69E+04	(K)VEGVA	9.20	4.60	0.87	632.87	5.97			
														3	15.7	7.21	93.3	1.89E+05	(R)AVPFV	9.38	6.05	0.98	701.73	4.65			
														2	12.83	12.83	81.2	8.59E+03	(R)AVPFV	9.37	0.00	0.63	1052.09	4.65			
18	1	1.2	5	5	79.28	8.2	5.78E+05	1.16E+05	69677.2	7.38	*Naja kaou*	Q91132	Cobra ven	2	12.37	12.37	70.7	1.17E+05	(R)IPIIDGI	5.70	11.19	1.00	464.26	4.21	1.60%	2.57%	0.04%
														2	16.4	11.47	85.8	2.53E+05	(K)GIYTP(	6.60	5.53	1.00	661.86	8.59			
														3	15.61	15.61	73.9	6.99E+04	(K)QLDIF\	7.53	5.40	0.99	462.91	5.21			
														2	17.28	17.28	88.7	7.06E+04	(K)YFTYL	7.80	5.40	0.97	587.83	8.50			
														2	17.62	12.68	91.6	6.78E+04	(-)ALYTLI	8.55	5.40	1.00	665.91	8.79			
18	2	2.1	13	10	162.9	22.8	6.77E+06	5.21E+05	58913.9	8.52	*Tropidolae*	gene22180_	L-amino a	3	10.26	5.09	70.8	1.99E+06	(R)VWEVI	5.77	0.13	0.99	461.93	6.04	7.20%	2.57%	0.18%
														2	18.21	14.96	92.6	1.22E+06	(K)SAGQL	5.40	0.13	0.99	641.32	4.53			
														2	18.36	15.17	94.1	5.46E+05	(R)SPLEE	5.42	5.53	0.99	519.24	4.53			
														2	16.88	9.14	94.6	4.48E+05	(R)VWEVI	5.77	0.13	0.99	692.39	6.04			
														2	13.39	8.05	80.5	8.14E+05	(K)FEPPL	5.90	44.25	0.94	462.76	6.00			
														2	10.35	3.49	73.5	1.85E+05	(R)IKFEPI	6.37	22.00	0.99	583.35	8.59			
														3	18.8	18.8	92.1	3.50E+05	(K)KDIQAI	6.43	5.40	0.98	566.30	8.50			
														2	19.14	12.29	93	9.23E+04	(K)KDIQAI	6.43	5.40	0.99	848.94	8.50			
														2	15.34	15.34	86.1	5.61E+05	(K)NAWYI	6.85	5.53	1.00	464.24	8.59			
														2	20.66	13.7	94.2	2.24E+05	(K)DIQAF(	7.15	0.00	0.99	784.89	5.83			
														3	12.43	12.43	70.3	4.53E+04	(K)DIQAF(	7.15	0.00	1.00	523.60	5.83			
														2	15.85	15.85	81.7	2.89E+04	(K)DCADI	8.88	5.40	0.92	1049.53	4.41			
														4	14.72	14.72	75.6	2.68E+05	(K)HVVIV(	10.28	6.15	1.00	908.74	5.32			
18	3	3.1	5	5	84.95	13.4	3.98E+06	7.96E+05	50974.4	9.34	*Tropidolae*	i04.contig1	Zinc metal	3	17.75	17.75	85.8	4.93E+04	(R)LYCVA	5.10	5.40	0.09	655.32	9.99	11.01%	2.57%	0.28%
														2	20.1	20.1	92.1	2.26E+06	(K)ASYLF	5.77	5.66	1.00	603.27	5.88			
														2	17.23	12.93	90.3	7.60E+05	(K)IIVQSS	5.95	5.66	0.99	650.38	5.84			
														2	13.37	13.37	86.6	8.82E+05	(K)LFAAW	6.48	5.40	1.00	382.22	9.75			
														3	16.5	16.5	88.8	2.76E+04	(R)IYNmA	9.42	4.46	0.96	583.32	8.75			
18	4	4.1	7	4	78.5	43.4	8.05E+06	1.15E+06	16330.1	6.31	*Tropidolae*	gene23090_	C-type lect	2	17.87	17.87	86.1	3.45E+06	(R)EQFE(	5.17	5.40	0.97	570.26	4.53	15.90%	2.57%	0.41%
														3	13.2	6.34	89.4	2.59E+06	(K)GSHLV	5.83	33.31	0.99	656.67	5.28			
														2	18.8	18.8	98.1	6.19E+05	(K)GSHLV	5.80	22.25	1.00	984.50	5.28			
														2	19.71	19.71	91.7	1.16E+06	(R)WSSYI	5.85	5.66	1.00	845.87	6.75			
														2	19.63	19.63	87.3	8.95E+04	(R)TFDN(	7.33	5.40	0.97	1213.51	4.21			
														3	22.12	22.12	90.8	9.51E+04	(R)TFDN(	7.95	0.00	0.99	804.01	4.21			
														2	18.02	18.02	72.4	4.96E+04	(R)TFDN(	7.95	0.00	0.90	1205.51	4.21			
18	5	5.1	5	4	71.34	20.7	1.06E+06	2.12E+05	27338.7	9.3	*Tropidolae*	gene19683_	GPX3	2	19.25	11.42	87.6	3.65E+05	(K)QEPG(	5.98	11.19	0.94	770.90	4.53	2.93%	2.57%	0.08%
														2	12.85	4.86	84.6	2.97E+05	(K)IYTFLK	6.48	5.40	1.00	392.73	8.59			
														3	17.17	17.17	100	7.79E+04	(K)HVRPG	7.60	0.00	0.98	643.35	11.00			
														2	16.8	10.47	92.6	2.80E+05	(R)LVILGI	8.33	5.40	1.00	746.90	8.75			
														3	22.07	22.07	96	3.85E+04	(R)LVILGI	8.33	5.40	0.91	498.27	8.75			
18	6	6.1	5	4	69.84	12.3	8.62E+05	1.72E+05	36683.3	9.32	*Tropidolae*	gene24659_	Snake ven	2	14.74	14.74	82.4	7.79E+05	(K)VGIIGY	5.68	0.13	1.00	476.28	8.56	2.38%	2.57%	0.06%
														2	21.51	16.64	95.7	4.06E+04	(K)ETPVL	7.68	0.00	0.97	859.94	4.53			
														3	15.85	12.07	77.2	1.83E+04	(K)ETPVL	7.68	0.00	0.92	573.63	4.53			
														3	15.84	8.13	87.7	1.71E+04	(K)YLGYL	8.97	3.63	0.98	652.70	5.96			
														2	17.75	17.75	86.5	7.01E+03	(K)YLGYL	9.28	0.00	0.98	722.89	4.21			
18	6	6.2	2	2	42.37	12.2	2.58E+05	1.29E+05	29358.2	7.3	*Tropidolae*	gene24655_	Snake ven	2	20.37	20.37	97.4	2.00E+05	(R)VVSLN	6.73	5.66	1.00	724.87	9.72	1.78%	2.57%	0.05%
														3	22	22	93.6	5.87E+04	(R)FHECN	8.23	0.00	0.98	803.38	4.54			
18	7	7.1	4	4	68.05	21.1	5.76E+05	1.44E+05	30421.9	5.14	*Tropidolae*	gene22208_	Gastric int	3	15.14	15.14	73.8	9.87E+04	(K)TKEEIE	4.57	3.88	0.98	590.96	4.57	1.99%	2.57%	0.05%
														2	14.35	1.18	86.2	3.39E+05	(K)EALILIT	6.70	5.66	1.00	450.79	6.10			
														2	22.06	13.18	97	8.94E+04	(K)LIGPYF	7.42	0.00	0.98	759.40	6.00			
														3	16.5	16.5	93.5	4.87E+04	(K)IPEGAI	8.73	5.40	0.90	718.73	6.00			
18	8	8.1	3	3	54.49	3.6	4.63E+05	1.54E+05	111620.8	5.55	*Tropidolae*	)71.contig1	Aminopept	2	16.17	9.1	89.2	1.83E+05	(K)TLFQC	6.63	5.66	0.98	498.77	9.41	2.13%	2.57%	0.05%
														2	17.57	17.57	88.6	9.35E+04	(R)ESALLI	6.97	0.00	0.99	900.44	4.37			
														2	20.75	20.75	83.1	1.86E+05	(R)AQIIDD	7.52	5.40	0.99	673.86	4.21			
18	9	9.1	3	3	48.89	16.3	5.83E+05	1.94E+05	26848	6.32	*Tropidolae*	gene21602_	Phosphodi	2	21.98	15.19	94.4	1.41E+05	(R)SQPTS	4.62	5.40	0.98	787.37	5.55	2.69%	2.57%	0.07%
														3	12.94	12.94	70.4	6.32E+04	(R)LWNYI	7.88	5.40	0.98	511.61	8.60			
														2	13.97	9.59	90.5	3.78E+05	(K)TFLPIF	9.47	6.31	1.00	630.85	5.18			
18	9	9.2	2	2	40.34	3.8	2.92E+05	1.46E+05	72248.1	8.91	*Tropidolae*	i48.contig1	Phosphodi	2	20.03	20.03	90.5	1.61E+05	(K)AATYF	7.12	5.40	1.00	678.34	6.04	2.02%	2.57%	0.05%
														2	20.31	14.93	95.1	1.31E+05	(R)VTEVL	8.97	3.63	0.99	707.40	4.37			
18	10	10.1	4	2	42.79	17	1.24E+07	3.10E+06	18686.1	9.4	*Tropidolae*	nigene18_T	Agglucetin	3	17.49	17.49	84.4	4.72E+06	(R)IINKPQ	4.90	5.40	0.97	500.60	6.07	42.87%	2.57%	1.10%
														2	18.24	12.46	93.4	1.05E+06	(R)IINKPQ	4.88	5.40	0.99	750.40	6.07			
														3	20.58	20.58	79.8	5.44E+06	(K)CPSEV	5.62	5.66	0.98	645.59	5.32			
														2	24.55	24.55	95.7	1.18E+06	(K)CPSEV	5.60	5.66	1.00	967.88	5.32			
18	11	11.1	3	2	33.67	9.8	3.74E+05	1.25E+05	36520.2	9.36	*Tropidolae*	gene20823_	Phospholi	3	17.24	17.24	96.3	3.10E+05	(R)KIPVP/	5.03	5.40	0.98	445.56	8.50	1.72%	2.57%	0.04%
														2	16.62	16.62	92.1	4.10E+04	(R)KIPVP/	5.87	0.13	0.89	667.84	8.50			
														3	16.43	16.43	95	2.30E+04	(K)HQGLF	8.08	5.40	0.89	746.70	5.32			
18	12	12.1	2	2	32.04	2.7	1.14E+05	5.70E+04	110664.5	5.54	*Gloydius t*	A5HUI5	Aminopept	3	13.39	13.39	72.9	3.43E+04	(R)YFGSL	7.33	5.40	0.99	517.96	8.50	0.79%	2.57%	0.02%
														2	18.65	18.65	84.4	7.95E+04	(R)AGFIDI	8.32	0.00	0.99	633.83	4.21			
18	13	13.1	2	2	28.2	10	2.19E+05	1.10E+05	23498.6	5.71	*Tropidolae*	256.contig1	Phospholi	2	16.74	10.87	92	2.31E+04	(R)NSGLV	8.53	5.40	0.98	572.83	9.75	1.51%	2.57%	0.04%
														2	11.46	11.46	74.9	1.96E+05	(K)GLLFLI	10.00	5.10	0.99	622.39	8.75			
								7.23E+06																	100.00%		

Supplementary File S2: Transcripts of Tropidolaemus wagleri venom proteins matched in LC-MS/MS protein identification

Phospholipase A2

>CL1260.contig1_NKM
Acidic phospholipase A2 Tgc-E6

```
      1 MRTLWIVAVLLVGVEGHLFQFETMIVKMTNRSGIFWYSSYGCYCGWGGRGQPQDPTDRCC          60
     61 FVHDCCYGKANGCDPKKDVYTYTMENGNIVCGGDTSCKNQVCECDKKAAVCFRNNLDTYN         120
    121 SKKYWMLSSKNCQD
```

>CL1723.contig1_TW
Basic phospholipase A2 homolog acutohaemolysin

```
      1 LFPVEGNLFDLWKMILQETGKNAFNYYGLYGCSCGVGGRRQPLDATDRCCFVHKCCYKKL          60
     61 TGCNPKMDHYSYIWTNKAIACGENDPCKNEMCECDKTLAICLGKNLDTYNKKYRNNVFLK         120
    121 FRCKKSSEQC
```

>CL1260.contig2_NKM
Phospholipase A2

```
      1 MRTLWIVAVLLVGVEGNLFDLWKMILQETGKNAFNYYGLYGCSCGVGGRRQPLDATDRCC          60
     61 FVHKCCYKKLTGCNPKMDHYSYIWTNKAIACGENDPCKNEMCE
```

Serine Protease

>CL448.contig1_TW
Serine protease 7

```
      1 VLIRVLATLLILQLSYAQKSSELVVGGSECNINEHRSLVVLFNSSRFLCSGILINQKWVI          60
```

61 TAAHCESENFQMQLGVHSKKVLNEDEQTRDPKKRFICPNRKNDDERDKDIMLIKLNEPIS 120
121 NSKHIAPLSLPSSPPSVGSVCRAMGWGTILPTKVKLPDVPHCVNIDLLDYSKCRAAYPDL 180
181 LAESSTLCAGILEGGKDTCVGDSGGPLICNREFQGIASWGSTICGYEYEPALYTKVFDHL 240
241 DWINSIIAGNTTATC

>CL448.contig2_TW
plasminogen activator-like protein precursor
1 VLIRVLATLLILQLSYAQKSSELVVGGSECNINEHRSLVVLFNSSRFLCSGILINQKWVI 60
61 TAAHCESENFQMQLGVHSKKVLNEDEQTRDPKKRFICPNRKNDDERDKDIMLIKLNEPIS 120
121 NSKHIAPLSLPSSPPSVGSVCRIMGWGSITSFK

>Unigene24832_TW
Beta-fibrinogenase mucrofibrase-3
1 MVLIRVLANLLILQLSYAQKSSELIIGGRPCNINEHRSLVLVYYDEFQCSGTLINQEWVV 60
61 SAAHCDGDNIEVQLGVHSKRLPNKDKQTRVAKEKYFCVSSKNYTKWNKDIMLIRLDSPVN 120
121 NSTHIAPVSLPSNPPSLGSVCRVMGWGSTTSPQETYPDVPHCANIKILDYAVCRAAHPWL 180
181 PATSRTLCAGILEGGKDSCKGDSGGPLICNEEIQGIVSWGWHPCAKQREPGHYTKVFDYT 240
241 DWIKNIMAGNLDATCP

>Unigene24831_TW
Snake venom serine protease gussurobin
1 MVLIRVLANLLILQLSYAQKSSELIIGGRPCNINEHRSLVLVYYDEFQCSGTLINQEWVV 60
61 SAAHCDGDNIEVQLGVHSKRLPNKDKQTRVAKEKYFCVSSKNYTKWNKDIMLIRLDSPVN 120
121 NSTHIAPVSLPSNPPSLGSVCRVMGWGSTTSPQETYPDVPHCANIKILDYAVCRAAHPWL 180
181 PATSRTLCAGILEGGKDSCKGDSGGPLICNGKFQAIVSWGGNPCGQRRKPGVYTKVFNYI 240
241 DWIQSIMAGNTTATCPP

C-type lectin

>Unigene14693_TW
Galactose-binding lectin
1 MGRCIFVSFGLLVVFLSLRGVKSSCCSNDSLPMNGLCYKIFNERKSWKDAEMFCRTYKPS 60
61 CHLASFHRSAESHDIAEYITDYHKGQAEVWIGLWDRKKDFSWEWTDRSCTDYLTWDKNQP 120
121 DHYQNKEFCVELVSSTGYLLWNDQVCESKNAFLCQCRF

>Unigene23090_TW
C-type lectin 9
1 LLVLFLSLRGAGAGLCCPMRWSSYEGHCYQVFKQEMNWTAAEKFCTQQHKGSHLVSVHNT 60
61 EEADFVVKMTHSSLGSTFVWIGVNNIWNGCHWKWSDGTALDYKEWREQFECLASRTFDNQ 120
121 WLSMDCSSTSPFVCKFQA

>Unigene18_TW
Agglucetin subunit alpha-2
1 MGRFIFVSFSLLVVFLCLSGTGADFKCPSEWYAYDQHCYRIINKPQTWGDAEKFCKKQAK 60
61 GGHLVSIQSSGEADFVAWMVTQNIETPFHYVWIGLRVQNKKKQCSTEWSDGSSVSYDNLL 120
121 ELYMRKCGGLEQEKGFRKWMNVACAQKHPFVCKFPPQC

Snake venom metalloproteinase

>Unigene330_TW
metalloproteinase isoform 1
1 VYPRKVTAVPKGAVQPKYEDAMQYEFKVNGDPVVLHLEKNKGLFSEGYSETHYSPDGREI 60
61 TTYPSVEDHCYYHGRIQNDADSTASISACNGLKGHFTLQGEMFLIEPLKLSDSEAHAIYK 120
121 YENVEKEDKAHKMCGVTQNWESYEPIQKASQLVVTPEQQRYLDAKKYVKFCVIVDHGMVQ 180
181 ENNGDLDKIKRIIYEIVNTMNEICIALNTRVALVCLEMWNNGDKINVTSAASVTLNSFRN 240
241 WKENDLMKRRSPDNAQLLTATDFDGDTVGLANIGSICDPKRSAAVVQYHSNISLLMAVTM 300
301 AHELGHNLGIHHDGDRCNCTVCIMSPRLSNHPSKEFSNCSKAEYQTYLINHRPQCILNKP 360
361 LKTDIISPPVCGNELLEVGEDCDCGSSENCQNPCCNAATCKLTPGSQCAEGLCCDQCRFK 420
421 RAGTECRAPKNDCDISENCTGRSAKCPTDLLQRNGQPCQNKLGYCYNGKCPIMTDQCIAL 480
481 WRPGVTVAPDVCFNYNLQGKDNFYCRRDNTTIFPCAQKDVKCGRLFCVEGPAVNGIVCKS 540
541 TTSRNDPDYGMVDLGSKCGDGKVCNSNRQCVDVKKAY

>CL1504.contig1_TW
Zinc metalloproteinase-disintegrin HV1
1 MCGVTQTNLESYEPIKKAAQSYLTPKQQKYLNAPKYMKLYLVADQIMYLKYGRNFTNLRT 60
61 RIYNMANILNLIFHRMNIHVAMLGLQVWSKEDKIIVQSSPDVTLKLFAAWRESVLLQNKS 120
121 HDNAQLLTGINFNGPTAGLAYLGGICNPMYSVAVVQDYNKIHHLVAIAMAHEMGHNLGMN 180
181 HDKDNCTCRAKSCVMAGTISCKASYLFSDCSREEHRAFLIKNMPQCILKKPLKTDIVSPP 240
241 VCGNYFVEVGEDCDCGSPATCRDPCCNPATCKLRQGAQCAEGLCCDQCRFKGAGTECRAA 300
301 KDECDMADLCTGRSAECIDRFQRDGQPCQNNYGYCYNGTCPTMRDQCISFFGPNAAVSQD 360
361 ACFQFNRLGNHYGYCRKEQNTKIACEPQNVKCGRLYCVASSPANKNPCNIRYSPYDEDTG 420
421 MVLPGTKCADGKACSNGKCI

L-amino acid oxidase

>Unigene22180_TW
L-amino acid oxidase
1 MNVFFMFSLLFLAALGSCAEDRSPLEECFRETDYEEFLEIARNGLKATSNPKHVVIVGAG 60
61 MAGLSAAYVLAEAGHQVTVLEASELVGGRVRTYRNEKEGWYANLGPMRLPEKHRIAREYT 120
121 RKFGLQLNEFFQENKNAWYFVKNIRKRVWEVNKDPGVLKYPVKPSEEGKSAGQLYEESLG 180
181 KVVEELQRTNCSYILNKYDTYSTKEYLIKEGNLSRGAVDMIGDLLNEDSGYYVSCIESMK 240
241 HDTIFAYEKRFHEIVGGMVQLPTSMYQAIKEKVHFNTRVIKIQQNVKEVTVTFQTSAKET 300
301 SSVTADYVIVCATSRATRRIKFEPPLPPKKAHALRSVHYRSGTKIFLTCTKKFWEDEGIH 360
361 GGKSTTDLPSRFIYYPNHNFTSGVGVIIAYGIGDDANFFQALDFKDCADIVFNDLSLIHQ 420
421 LPKKDIQAFCYPSVIQKWSLDKYAMGGITTFTPYQFQHFSEPLTAPFGRIYFAGEYTAQA 480
481 HGWIDSTIKSGLKAARDVNRASENPSGIHLSNDNKL

5' nucleotidase

>Unigene24659_TW
Snake venom 5'-nucleotidase
1 MQTPKRRRGAQACPRSSPSPLLLLGAVWFCAALSVAAGSFELTLLHTNDVHARVEQTSRD 60
61 SGKCTGQDCYGGVARRATKIRELRAKHRHVLLLDAGDQYQGTIWFSYFKGREVVKFMNSL 120
121 RYDAMALGNHEFDNGLAGVLDPLLKHANFPILSANIKPKGPIASNISGYILPYKIINVGS 180
181 EKVGIIGYTTKETPVLSNPGPYLEFRDEVEELQKHANKLTTLGVNKIIALGHSGFLEDQR 240
241 IARKVKGVDVVIGGHTNTFLYTGSPPSTEVAAGNYPFMVQSDDGRQVPVVQAYAFGKYLG 300
301 YLIVVFDDKGNVIKASGNPILLNKDIPEDQDVK

>Unigene24655_TW
Snake venom 5'-nucleotidase
1 IPEDQDVKAEVNKMKIQLHNYSSQEIGKTIVYLNGTTQACRFHECNLGNLICDAVVYDNV 60
61 RHTDDKEWNHVSMCIVNGGGIRSPIDERANNGTITLEELTAVLPFGGTFDLLQIKGYALK 120
121 QAFEHSVHRHGQGMGELLQVSGIKVVYDLSRKPGSRVVSLNVLCTQCRVPAYVPLEMKKT 180
181 YKLLLPSFLASGGDGYHMLKGDSSNHTSGNLDISIVGDYIKRMGKVFPAVESRMIFSAGT 240
241 LFQAQLFLTWGLCISLLYFIL

>Unigene10279_CRM
Snake venom 5'-nucleotidase
1 MQTPKRGTPGCPPSCPSPPPPLLVGAVWFCALLSLAAASFELTLLHTNDVHARVEQTSRD 60
61 SGKCTGQECYGGVARRATKIRELRAKHRHVLLLDAGDQYQGTIWFNFFKGREVVTFMNRL 120
121 RYDAMALGNHEFDNGLAGVLDPLLKYASFPILSANIIPKGPIASNISGYILPYKIINVGS 180
181 EKVGIIGYTTKETPVLSNPGPYLEFRDEVEELQKHVNKLTTLGVNKIIALGHSGFLEDQR 240
241 IAQKVKGVDVVIGGHTNTFLYTGTPPSTEVAAGNYPFMVQSDDGRQVPVVQAYAFGKYLG 300
301 YLNVIFDDKGNVIKASGNPILLNKDIPEDQDVKTEVNKMKIQLHNYSSQEIGKTIVYLNG 360
361 TTHACRFHECNLGNLICDAVIYDNVRHPDDNEWNHVSMCIINGGGIRSPIDERANNGTIT 420
421 LEELTAVLPFGGTFDLLQVKGDALKQAFEHSVHRHGQGTGELLQTSGIKVVYDLSRRPGM 480
481 RVVSLKVLCTECRVPTYVPLETGKTYKLLLPSFLASGGDGYHMLKGDASNHSSGNLDISI 540
541 VGDYIKRMGKVFPAVEGRMIFSAGTLFQAQLFLTWALCISLLYFIL

Phosphodiesterase

>CL2548.contig1_TW
phosphodiesterase 1
1 MIQQKVLFISLVAVALGLGLGLGLKESVQPQVSCRYRCNETFSKMASGCSCDDKCTERQA 60
61 CCSDYEDTCVLPTQSWSCIKLRCGEKRIANALCSCSEDCLEKKDCCTNYKSICKGETSWL 120
121 KDKCASSIATQCPAGFEESPLILFSMDGFRAGYLESWDSLMPNINKLKTCGTHAKYMRAV 180
181 YPTKTFVNHYTIATGLYPESHGIIDNNIYDVNLNLNFSLSGSTAKNPAWWGGQPIWHTAT 240
241 YQGLKAATYFWPGSEVKINGSYPTIFKNYNKSISFEARVTEVLTWLDLPKAKRPDFYTLY 300
301 IEEPDTTGHNYGPVSGEIIKALQMADRTLGMLMEGLKQRNLHNCINLILLADHGMEEISC 360
361 DRLEYMANYFNNVDFFMYEGPAPRIRSKNVPKDFYTFDSEGIVKNLTCKKPKQYFKAYLS 420
421 KDLPKRLHYVNNVRIDKVNLMVDQQWMAVRNKKFTRCKGGTHGYDNEFKSMQAIFLAHGP 480

```
481 GFKEKNEVPSFENIEVYNLMCDLLKLKPAPNNGTHGSLNHLLKNPFYTPSPAKEQSSPLL   540
541 CPFGPVPSPDVSGCNCSSITDLEKVNQRLNLNNQAKTESEAHNLPYGRPQVLQNHSNYCL   600
601 LHQAKYISAYSQDILMPLWSSYTIY
```

>Unigene21602_TW
phosphodiesterase 1

```
  1 WSSYTIYRSQPTSAPPSASDCLRLDVRIPAAQSQTCSNYQPDLTITPGFLYPPNFNLSNF    60
 61 EQYDALITSNIVPMFKGFTRLWNYFHTTLLPKYASERNGLNVISGPIFDYNYDGHFDSYS   120
121 TIKEHVKNTKIPIPTHYFVVLTSCENQINTPLNCLGPLKVLSFILPHRPDNSESCADTLP   180
181 ENLWVEKRIQIHTARVRDVELLTGLNFYSGLQQPLPETLQLKTFLPIFVNPVN
```

Cysteine-rich secretory protein

>CL1654.contig2_TW
cysteine-rich seceretory protein Og-CRPa

```
  1 MIAFIVLPILAVVLQQSSGSVDFDSESPRNPEIQNEIIDLHNSLRRSVNPTASNMLKMEW    60
 61 YPEAAANAERWAYRCIEEHSSRNSRVVEEINCGENIYMSSNPMKWTEIIHAWYGEYKNFK   120
121 YAVGADPSNAVIGHYTQIVWYKSYLAGCAAAYCPSSEYSYFYVCQYCPEGNIIGKSAFPY   180
181 TSGPPCGDCPSACDYGLCTNPCTEKDIYINCNSLVQQSGCQDKRMQSYCPASCFCQNKII   240
```

Cobra venom factor

>Unigene370_NSM
Cobra venom factor

```
   1 MEGMALYLVAALLIGFPGSSHGALYTLITPAVLRTDTEEQILVEAHGDNTPKQLDIFVHD    60
  61 FPRKQKTLFQTRVDMNPAGGMLVTPTIEIPANEVSTDSRQNQYVVVQVTGPQVRLEKVVL   120
 121 LSYPSSFLFIQTDKGIYTPGSPVLYRVFSMDHNTSKMNKTVIVEFQTPEGILVSSNSVDL   180
 181 NFFWPYNLPDLVSLGTWRIVAKYEHSPENYTAYFDVRKYVLPSFEVRLQPSEKFFYIDGN   240
 241 ENFHVSITARYLYGEEVEGVAFVLFGVKIDDAKKSIPDSLMRIPIIDGDGKATLKRDTFR   300
 301 SRFPNLNELVGHTLYASVTVMTESGSDMVVTEQSGIHIVTSPYQIHFTKTPKYFKPGMPY   360
 361 ELTVYVTNPDGSPAAHVPVVSEAIHSEGTTLSDGTAKLNLNTPLNAQSLPITVRTNHGDL   420
 421 PRERQAIKSMTATAYQTQGGSGNYLHVAITSTEIKPGDNLPVNFNVKGNANSLNQIKYFT   480
 481 YLILTKGKIFKVGRQPRGDGQNLVTMNLHITPDLIPSFRFVAYYQVGNNEIVADSVWVDV   540
 541 KDTCMGTLVVKGASSRDNRIQKPGAAVKIKLEGDPGARVGLVAVDKAVYVLNDKYKISQA   600
 601 KIWDTIEKSDFGCTAGSGQNNLGVFEDAGLALTTSTNLNTKQRSAAKCPQPANRRRRSSV   660
 661 LLLDSKASKAAEFQDQDLSKCCEDVMHENPMGYTCEKRAKYIQEGDACKAAFLECCRYIK   720
 721 GVRDENQRESELFLARDDNEDGFIADSDIISRSDFPESWLWLTKDLTKEPNSQGISSKTM   780
 781 SFYLRDSITTWVVLAVSFTPTKGICVAEPYEIRVMKVFFIDLQMPYSVVKNEQVEIRAIL   840
 841 HNYVNEDIYVRVELLYNPAFCSASTKGQRYRQQFPINALSSRAVPFVIVPLEQGLHDVEV   900
 901 TASVRGELASDGVRKKLKVVPEGVQKSIVTIIKLDPRAKGVGGTQREVVKARKLDDKVPD   960
 961 TEIETKIIIQGDPVAQIIENSIDGSKLNHLIITPSGCGEQNMIRMTAPVIATYYLDTTEQ  1020
1021 WETLGINRRTEAVNQIMTGYAQQMVYKKADHSYAAFTNRASSSWLTAYVVKVFAMAAKMV  1080
1081 AGISHEIICGGVRWLILNRQQPDGAFKENAPVLSGTMQGGIQGAEEEVYLTAFILVALLE  1140
1141 SKTICNDYVNSLDSSIKKATNYLLKKYEKLQRPYTTALTAYALAAADQLNDDRVLMAAST  1200
1201 GRDHWEEYNAHTHNIEGTSYALLALLKMKKFDQTGPIVRWLTDQNFYGGTYGQTQATVMA  1260
1261 FQALAEYEIQMPTHKDLNLDITIELPDREVPIRYRINYENALLARTVETKLNQDFTVTAS  1320
1321 GDGKATMTILTFYNAQLQEKANVCNKFHLNVSVENIHLNAMGASGALMLKICTRYLGEVD  1380
1381 STMTIIDISMLTGFLPDAEDLTRISRGVDRYISRYEVDNNMAQKVAVIIYLDKVSHSEDE  1440
1441 CLQFKILKHFEVGFIQPGSVKVYSYYNLDEKCTKFYHPDKGTGLLNKICVGNVCRCAGET  1500
1501 CSSLNHQERIDVPLQIEKACETNVDYVYKTKLLRIEEQDGNDIYVMDVLEVIKQGTDENP  1560
1561 RAKTHQYISQRKCQGALNLKVNDDYLIWGSRSDLLPTKDKISYIITKNTRIERWPHEDEC  1620
1621 QEEEFQKLCDDFAQFSFTLTVFGCPT
```

>CL4560.contig1_NSM
Cobra venom factor

```
   1 MEGMALYLVAALLIGFPGSSHGALYTLITPAVLRTDTEEQILVEAHGDNTPKQLDIFVHD    60
  61 FPRKQKTLFQTRVDMNPAGGMLVTPTIEIPAKEVSTDSRQNQYVVVQVTGPQVRLEKVVL   120
 121 LSYQSSFLFIQTDKGIYTPGSPVLYRVFSMDHNTSKMNKTVIVEFQTPEGILVSSNSVDL   180
 181 NFFWPYNLPDLVSLGTWRIVAKYEHSAENYTAYFDVRKYVLPSFEVRLQPSEKFFYIDGN   240
 241 ENFHVSITARYLYGKKVEGVAFVLFGVKIDDAKKSIPDSLTRIPIIDGDGKATLKRDTFR   300
 301 SRFPNLNELVGHTLYASVTVMTESGSDMVVTEQGGIHIVTSPYQIHFTKNPKYFKPGMPY   360
 361 ELTVYVTNPDGSPAAKVPVVSEAFHSMGTTLSDGTTKLILNTPLNAQSLPITVRTNHEVF   420
 421 PRERQATKSMTATAYQTQGGSGNYLHVAITPTEIKPGDNLPVNFNVRGNANSLKQIKYFT   480
 481 YLILNKGKIFKVGRQPRRDGQNLVTMNLHITPDLIPSFRFVAYYQVGNNEIVADSVWVDV   540
 541 KDTCMGTLVVKGDNLIQMPGAAMKIKLEGDPGARVGLVAMDKAVYVLNDKYKISQAKIWD   600
 601 TIEKSDFGCTAGSGQNNLGVFEDAGLALTTSTNLNTKQRSAAKCPQPANRRRRSSVLLLD   660
 661 SKASKAAEFQDQDLRKCCEDVMHENPMGYTCEKRAKYIQEGDACKAAFLECCRYIKGVRD   720
 721 ENQRESELFLARDDNEDGFIPDSDIISRSDFPESWLWLTKDLTEEPNSQGISSKTMSFYL   780
 781 RDSITTWVVLAVSFTPTKGICVAEPYEIRVMKVFFIDLQMPYSVVKNEQVEIRAILHNYV   840
 841 NEDIYVRVELLYNPVFCSASIKGQRYQQQFPIKALSSRAVPFVIVPLEQGLHDVEIKASV   900
 901 REALWSDGVRKKLKVVPEGVQKSIVTIVKLDPRAKGVGGTQREVVKARKLDDRVPDTEIE   960
 961 TKIIIQGDPVAQIIENSIDGSKLNHLIITPSGCGEQNMIRMAAPVIATYYLDTTEQWETL  1020
1021 GINRRTEAVNQIMTGYAQQMVYKKADHSYAAFTNPASSSWLTAYVVKVFSMAAKMVAGIS  1080
1081 HEIICGGVRWLILNRQQPDGAFKENAPVLSGTMQGGIQGAEEEVCLTAFILVALLESKTI  1140
1141 CNDYVNSLDSSIKKATNYLLKKYEKLQRPYTTALTAYALAAADQLNDDRVLMAASTGRDH  1200
1201 WEEYNAHTHNIEGTSYALLALLKMKKFDQTGPIVRWLTDQNFYGGTYGQTQATVMAFQAL  1260
1261 AEYEIQMPTHQDLNLDITIELPDREVPIRYKINYENALLARTVETKLNQDFTVTASGDGK  1320
1321 ATMTILTFYNAQLQEKANVCNKFHLNVSVENIHLNAMGAKGALMLKICTRYLGEVDSTMT  1380
1381 IIDISMLTGFLPDAEDLTRLSKGVDRYISRYEVDNNMAQKVAVIIYLNKVSHSEDECLKF  1440
1441 KILKHFEVGFIQPGSVKVYSYYNLDEKCTKFYHPDKGTGLLNKICIGNVCQCAGETCSSL  1500
1501 NHQERIDVPLQIEKACETNVDYVYKTKLLRIEEQDGNDIYVMDVLEVIKQGTDKNPR     1560
```

Phospholipase B

>Unigene20823_TW
Phospholipase B

```
  1 LPGYENIYFAHSSWFTYAATLRIYKHLDFKITDPQTKTGRASFSSYPGLLVSLDDFYILG    60
 61 SGLIMLQTTNSVFNLSLLKKVVPESLFAWERVRIANMMADSGKTWAETFEKQNSGTYNNQ   120
121 YMILDTKKIKLQRSLEDGTLYIIEQVPKLVKYSDQTKVLRNGYWPSYNIPFHKVIYNMSG   180
181 YGEHVQRYGLEFSYEMAPRAKIFRRDQGKVTDMESMKSIMRYNNYKEDPYAKRNPCNTIC   240
241 CRQDLDRKIPVPAGCYDSKVADISMAAKFTAYAINGPPVEKGLPVFSWIHFNKTKHQGLP   300
301 ESYNFDFVTMKPVL
```

Aminopeptidase

>CL3071.contig1_TW
aminopeptidase N TW1b

```
  1 MAKGFFISKSLGVVGLVLGLGAVATIIALSVVYSQEKQRADDLSDQVPSSTSAAVAPGTT    60
 61 IAGTAQATTANGTAQATTTKPLEPWDLMRLPKTLMPTFYTISLQPFLTEISPNFYIFKGN   120
121 STVEFQCKQPTDLILIHSKKLNYTLQGAFFVSLTGTGVAPPAIHNTWLEEKTEYLVVKLK   180
181 ENLQQNKNYQLHAVFTGELADDLAGFYRSAYTEEGVPKLLATTQMQAADARKAFPCFDEP   240
241 AMKANFSITLIHLPAYKALSNMPIKSAEKVTMPDGEIWTRTEFHPTLKMSTYLLGFIVSE   300
301 FENVSVIENNTLIQIWGRPKAIREGQGNYALNVTGTILRFFEREYRVPYPLTRLDQVALP   360
361 DFNAGAMENWGLITYRESALLFDQQYSSIGNKERIVTVIAHEVSHQWFGNLVTLEWWNEL   420
421 WLNEGFASYVEYLGAHEAESTWNIKDLIVPNDVYRVMAIDALASSHPLSTPADEINTPAQ   480
481 ISEVFDSISYSKGASVIRMLSEFLTEAVFREGLQTYFETYQYGNTVCDDLWEQLQKAVSK   540
541 NNVNLPSTVKTIMDRWTLQMGFPVLTVNTSAGIISQRHFLLDPESPVERPSPFHYIWIVP   600
601 VSWLSKGKQAEMYWLTDISAKNDNFSASADPTQWLLLNINVTGYFRVNYDLENWQRLMNQ   660
661 LNEDLQMIPVLNRAQIIDDAFNLARAKHVGSDLALNTTRYLAQEREYLPWDTALGNLDYF   720
721 RLMFDRSEVYGPMQKYIRKQVTPLFEYFQKLTFNWTKIPDGLMNQYSQILAIRTACSYGV   780
781 PGCNELASSWFEAWKNNHSSNPISANLRSAVYCSAIRTGSLEDWDFAWEMFRQAPVISEA   840
841 DKLRAALTCSQTPWILQRYLQFTLDPSMIRRQDAMSTINSIASNVVGQSLAWDFVRMNWK   900
901 TLFQQFGRSSFSFSSLIQSVTQRFSSPFELQQLEQFKKDNADVGFGSATRALEQALERTK   960
961 ANIKWVAENKALVLKWFQDNS
```

Phospholipase A_2 inhibitor

>CL2256.contig1_TW
phospholipase A2 inhibitor beta TW1b

```
  1 LSLSENQLAELDPSWFETLEDLKILRLDHNELKEIPGPIFQKLTKLLSLDLSSNLLHCLS    60
 61 PKMFKGLLFLNVLNLKNNPIRSIAPNTFHRNSGLWVLSLRNCSLTHVPAGLFQPLNHLGR   120
121 LDLSANEITALEPSLLDVSSEFTLDLSGNPWACDCRMQALLNWVQGGDVDLFSKEDVVCA   180
181 FPKSLNGQVATSLKGSQLCPCUSFRTGLF
```

Non-toxin

>Unigene19683_TW
GPX3

```
      1 LKGAWILSLLLAGLFQPNLGQEQQKILTGLQLSHQHDGARSCLHAQPIPDRAKVDCYDSV     60
     61 QGSAHDYGALSLTGEYIPFRNYDGKYILFVNVATYUGLTGQYLELNALQSSLGIDRLVIL    120
    121 GFPCNQFGKQEPGQNSEILQGIKHVRPGGGFVPNFQLFQKGDVNGENEQRIYTFLKNSCP    180
    181 PVVESFGDPAKLFWSPMKLHDIKWNFEKFLVNPQGKPVMRWFHRTNVSSVKNDIIRYMRK    240
    241 N
```

>Unigene22208_TW
gastric intrinsic factor-like

```
      1 QVLEEKTKEEIEHIDTTETPKTTYYQLALDTLALCIEKSPELDRAATALAKAALTNRFQF     60
     61 NGRLSVDTAAVASLALFCVHEGRISSQQSKLTATIKEALILITKQILNEQQTNGLLGNIY    120
    121 STGLAMQALNVTSEFYSDNPWNCPKTLNEVLSKIPEGAFSTPAAASQILPSLVGKTYLDV    180
    181 SNVACTYETVTVGYKVKNKLIGPYFEYSTTVKVPKDSVLLAVLEAARQFNAKKFSFQTEE    240
    241 TSWGPMVISISGLQANTNDKTYWQFLSDETPLEQ
```

>CL3502.contig1_TW
intestinal-type alkaline phosphatase 1-like TW1b

```
      1 AVIPVEEEKPSFWNELADKAIRKASRLQYRNYQAKNLILFLGDGMGVPTITATRILKGHM     60
     61 ENPPSPESPLSMDSFPYVALSKTYNVDRQVPDSAGTATAYLCGVKGNYGTIGLSAAARYN    120
    121 QCNTTAGNEVVSVLKRAQLAGKSVGIVTTTRVQHASPAGNYAHVVNRNWYSDASLPQSAL    180
    181 EGGCKDIALQLLENVDLTVVLGGGRKYMTPQGTPDPEYPTQNSSRGVRKDNRNLIEEWLR    240
    241 ASPGNKYVWNRIDLLQAVSDPKVHRLMGLFEPSDMKYELYRDNTTDPSLAEMTEAAIKLL    300
    301 SSNPKGFYLFVEGGRIDHGHHDGIAKRALTEAIEFDKAIERAGKLTREEDTLTAVTADHS    360
    361 HVFSFGGYTLRGASIFGLAPGKGIDGKAYTSIVYGNGPGYQITDKGRPDVTAEESENDTY    420
    421 HQQAAVPLVSETHGGEDVAIMAKGPMAHLFHGVQEQSYIAHVMAYASGLEPYNLRNYYSA    480
    481 TSAAAPKAGLACLFLVPLLLW
```

>CL2565.contig1_TW
ADP-ribosyl cyclase 1

```
      1 MPFQNSSSWTKKQKLILTGVIVVLLGTLTVFVVFRLFRLGRKKIPIAEEQEWKGRGTTEH     60
     61 LLEIVLGRCYNFINTINPELRNKDCLKVWKLFEQAFLYKDPCRVTEEDYQPLMDLARYSI    120
    121 PCNKSLFWSKTYDLAHHYTKTNNDFLTLEDTLLGYIADGISWCGNPSDSGVNYESCPKWT    180
    181 ECENNPSSIYWKLASKMFAETSCGTVQVMLNGSTMSEAFRKSSIFGSVEIVNLNPKEVSK    240
    241 MQIWLMHDIDGPQRESCTGHSIAQLRLREILENRNISVSCEDNYRPARLLQCTRNPNHIVCK    300
```
